# Quiescent optical solitons with complex Ginzburg–Landau equation having a dozen forms of self–phase modulation

**DOI:** 10.1016/j.heliyon.2023.e15661

**Published:** 2023-04-22

**Authors:** Ahmed H. Arnous, Anjan Biswas, Yakup Yıldırım, Luminita Moraru, Simona Moldovanu, Abdulah A. Alghamdi

**Affiliations:** a Department of Physics and Engineering Mathematics, Higher Institute of Engineering, El–Shorouk Academy, Cairo, Egypt; b Department of Mathematics and Physics, Grambling State University, Grambling, LA, 71245, USA; c Mathematical Modeling and Applied Computation (MMAC) Research Group,Department of Mathematics, King Abdulaziz University, Jeddah, 21589, Saudi Arabia; d Department of Applied Mathematics, National Research Nuclear University, 31 Kashirskoe Hwy, Moscow, 115409, Russian Federation; e Department of Applied Sciences, Cross–Border Faculty of Humanities, Economics and Engineering,Dunarea de Jos University of Galati, 111 Domneasca Street, Galati, 800201, Romania; f Department of Mathematics and Applied Mathematics,Sefako Makgatho Health Sciences University, Medunsa, 0204, South Africa; g Department of Computer Engineering, Biruni University, 34010 Istanbul, Turkey; h Department of Chemistry, Physics and Environment, Faculty of Sciences and Environment, Dunarea de Jos University of Galati, 47 Domneasca Street, 800008 Galati, Romania; i Department of Computer Science and Information Technology, Faculty of Automation, Computers, Electrical Engineering and Electronics, Dunarea de Jos University of Galati, 47 Domneasca Street, 800008 Galati, Romania

**Keywords:** Kudryashov, Nonlinear chromatic dispersion, Solitons

## Abstract

The current study focuses on the recovery of quiescent optical solitons through the use of the complex Ginzburg–Landau equation when the chromatic dispersion is rendered to be nonlinear. A dozen forms of self-phase modulation structures are taken into consideration. The utilization of the enhanced Kudryashov's scheme has led to the emergence of singular, dark, and bright soliton solutions. The existence of such solitons is subject to certain parametric restrictions, which are also discussed in this paper.

## 1 Introduction

The golden jubilee celebration of solitons, since the emergence of their concept in 1973, is underway. Thus, five decades of soliton science and technology have been sculpted into their current engineering marvel, and a lot is yet to be covered. One of the features that is less addressed is the accidental rendering of the nonlinearity of chromatic dispersion (CD). The soliton transmission technology is based on the delicate balance that exists between self–phase modulation (SPM) and linear CD. Often, it so happens that the linear CD comes out as nonlinear that initiates a global catastrophe. This can be triggered from a complete spectrum of sources such as random injection of pulses at the initial end of the fiber, rough handling of optical fibers and others. The solitons gets stalled during their transmission across trans–oceanic and trans–continental distances since they transform into quiescent solitons [[1], [2], [3], [4], [5], [6], [7], [8], [9], [10], [11], [12]]. This, therefore, needs to be addressed and appropriate measures and means must be taken into account to prevent the occurrence of such a catastrophic situation.

A dozen forms of SPM structures are used to address the complex Ginzburg–Landau equation (CGLE) in order to derive quiescent solitons, as presented in this paper. The reason for studying this model is that CGLE is a generalized version of the most fundamental model, namely the nonlinear Schrödinger's equation (NLSE) that is derived from Maxwell's equation in Electromagnetic Theory by the usage of multiple scales perturbation terms. The integration mechanism in the current paper is the enhanced Kudryashov's technique. This approach would lead to quiescent bright, singular and dark optical solitons. The existence of such stationary solitons is guaranteed for specific parameter constraints for each form of SPM. These constraints are also presented for each of the nonlinear refractive index structures. The full set of findings are secured and displayed after succinct pen-picture of the adopted Kudryashov's scheme.

The results of the study of quiescent solitons with nonlinear CD were first reported during 2006. Later, this study gained a lot of attention and additional models from fiber optics were handled to look at stationary solitons. Those models are Lakshmanan–Porsezian–Daniel equation, Sasa–Satsuma model, NLSE amongst others. CGLE with a dozen forms of refractive index structures has been lately addressed using a variety of mathematical approaches. The generalized $G^{\prime }/G$–expansion method yielded a variety of stationary solitons during 2022 [3,9]. During the same year a direct software approach led to the emergence of quiescent solitons for CGLE, although implicit [2].

## 2 The enhanced Kudryashov's approach

Let us take a look at the following representation of the nonlinear evolution equation

(1) $$G\left(u,u_{x},u_{t},u_{xt},u_{xx},...\right)=0\text{.}$$

The polynomial function G depends on u and its time and space independent variables, where u=u(x,t) is the unknown function it represents. By employing the transformation

u(x,t)=U(ξ),ξ=μ(x−υt),

where the transformation of equation (1) into an ordinary differential equation (ODE) is achieved by making μ and υ variables, as shown below

(2) $$P\left(U,-\mu \upsilon U^{\prime },\mu U^{\prime },\mu^{2}U^{\prime \prime },...\right)=0\text{.}$$

Step–1: The solution to (2) is stated as

(3) $$U\left(\xi \right)=\lambda_{0}+\sum_{l=1}^{N}\;\sum_{i+j=l}\;\lambda_{ij}Q^{i}\left(\xi \right)R^{j}\left(\xi \right)\text{,}$$

where constants $\lambda_{0}$ and $\lambda_{ij}$ (where i and j are integers between 0 and N) are given, and Q(ξ) and R(ξ) are functions that satisfy the following ODEs

(4) $$R^{\prime }{\left(\xi \right)}^{2}=R{\left(\xi \right)}^{2}\left(1-\chi R{\left(\xi \right)}^{2}\right)\text{,}$$

and

(5) $$Q^{\prime }\left(\xi \right)=Q\left(\xi \right)\left(\eta Q\left(\xi \right)-1\right)\text{.}$$

One can apply the following formulas to determine the solutions to equations (4), (5), as presented below

(6) $$R\left(\xi \right)=\frac{4c}{4c^{2}e^{\xi }+\chi e^{-\xi }}\text{,}$$

and

(7) $$Q\left(\xi \right)=\frac{1}{\eta +de^{\xi }}\text{,}$$

where c,d,η and χ are arbitrary constants.

Step–2: The positive integer value N in Eq. (3) can be found by balancing the nonlinear term with the highest order derivative in Eq. (2).

Step–3: The insertion of (3), (4), (5), (2) generates a polynomial in terms of R(ξ), $R^{\prime }\left(\xi \right)$, and Q(ξ). All terms with the same power are set to zero to produce an over-determined algebraic equation system, which can be addressed by the aid of Mathematica to determine the constants k, v, c, d, η, χ, $\lambda_{0}$, and $\lambda_{ij}$. As a result, we secure the solutions of Eq. (1).

## 3 CGLE with nonlinear CD

The main governing equation reads

(8) $$iq_{t}+a{\left({\left|q\right|}^{n}q\right)}_{xx}+F\left({\left|q\right|}^{2}\right)q=\frac{1}{{\left|q\right|}^{2}q^{*}}\left\{\alpha {\left|q\right|}^{2}{\left({\left|q\right|}^{2}\right)}_{xx}-\beta {\left\{{\left({\left|q\right|}^{2}\right)}_{x}\right\}}^{2}\right\}+\gamma q\text{.}$$

Here in (8), the function q(x,t), which is complex valued, is the dependent variable that is determined by the independent variables x and t, representing the spatial and temporal components, respectively. Then, $i=\sqrt{-1}$. The physical parameters are described as given here. The linear temporal evolution of the solitons is indicated by the first term. The nonlinear CD, with n as the nonlinearity parameter, is indicated by the coefficient of a. The linear CD is indicated by the coefficient of a when the nonlinearity parameter n equals zero. The structure of the nonlinear refractive index is represented by the functional F. A dozen variations of F will be explored in the paper. The terms of perturbation are represented by α and β while γ stands for the detuning effect. To obtain stationary solitons, we introduce the split

(9) $$q\left(x,t\right)=U\left(kx\right)e^{i\left(\omega t+\theta_{0}\right)}\text{,}$$

where the phase constant gives rise to $\theta_{0}$, and ω is the symbol for the wave number. On substituting (9) into (8), we get the following equation

(10) $$ak^{2}\left(n+1\right)U^{n+1}U^{\prime \prime }+ak^{2}n\left(n+1\right)U^{n}{U^{\prime }}^{2}+F\left(U^{2}\right)U^{2}-2\alpha k^{2}UU^{\prime \prime }-2k^{2}\left(\alpha -2\beta \right){U^{\prime }}^{2}+\left(\gamma -\omega \right)U^{2}=0\text{.}$$

The following subsections will discuss Eq. (8) with different types of SPM structure.

### 3.1 Kerr law

Eq. (8) appears as

$$iq_{t}+a{\left(|q{|}^{n}q\right)}_{xx}+b|q{|}^{2}q=\frac{1}{|q{|}^{2}q^{*}}\left\{\alpha |q{|}^{2}{\left(|q{|}^{2}\right)}_{xx}-\beta {\left\{{\left(|q{|}^{2}\right)}_{x}\right\}}^{2}\right\}+\gamma q\text{.}$$

This leads to the transformation of Eq. (10) into

(11) $$ak^{2}\left(n+1\right)U^{n+1}U^{\prime \prime }+ak^{2}n\left(n+1\right)U^{n}{U^{\prime }}^{2}+bU^{4}-2\alpha k^{2}UU^{\prime \prime }-2k^{2}\left(\alpha -2\beta \right){U^{\prime }}^{2}+\left(\gamma -\omega \right)U^{2}=0\text{.}$$

For integrability, we set n=1. So Eq. (11) changes to

(12) $$2ak^{2}U^{2}U^{\prime \prime }+2ak^{2}U{U^{\prime }}^{2}+bU^{4}-2\alpha k^{2}UU^{\prime \prime 2}\left(\alpha -2\beta \right){U^{\prime }}^{2}+\left(\gamma -\omega \right)U^{2}=0\text{.}$$

Balancing $U^{2}U^{\prime \prime }$ with $U^{4}$ in Eq. (12) gives N=2. Thus, we arrive at

(13) $$U=\lambda_{0}+\lambda_{01}R+\lambda_{10}Q+\lambda_{11}RQ+\lambda_{02}R^{2}+\lambda_{20}Q^{2}\text{.}$$

Inserting (13) along with (4), (5), (12) paves way to a polynomial of Q,R and $R^{\prime }$. All terms with the same powers are gathered and set to zero, resulting in a system of equations. Solving this system leaves us with the outcomes:

**Result–1**:

$$\lambda_{0}=\lambda_{01}=\lambda_{10}=\lambda_{11}=\lambda_{20}=0,\lambda_{02}=\frac{\chi \left(4\beta -5\alpha \right)}{4a},k=\pm \frac{\sqrt{b\left(4\beta -5\alpha \right)}}{4\sqrt{5}a}\text{,}$$

(14) $$\omega =\frac{5a^{2}\gamma +b\left(\alpha -\beta \right)\left(5\alpha -4\beta \right)}{5a^{2}}\text{.}$$

Substituting (14) along with (6), (13) yields the optoelectronic wave field

$$q\left(x,t\right)=\frac{\chi \left(4\beta -5\alpha \right)}{4a}{\left(\frac{4c}{4c^{2}\;\exp\left[\pm \frac{\sqrt{b\left(4\beta -5\alpha \right)}}{4\sqrt{5}a}\;x\right]+\chi \;\exp\left[\mp \frac{\sqrt{b\left(4\beta -5\alpha \right)}}{4\sqrt{5}a}\;x\right]}\right)}^{2}$$

(15) $$\times e^{i\left(\left\{\frac{5a^{2}\gamma +b\left(\alpha -\beta \right)\left(5\alpha -4\beta \right)}{5a^{2}}\right\}t+\theta_{0}\right)}\text{.}$$

The emergence of the quiescent bright and singular solitons can be observed by setting $\chi =\pm 4c^{2}$ in equation (15), as indicated below

$$q\left(x,t\right)=\pm \frac{4\beta -5\alpha }{4a}\;\sec\;h^{2}\left[\frac{\sqrt{b\left(4\beta -5\alpha \right)}}{4\sqrt{5}a}\;x\right]\;e^{i\left(\left\{\frac{5a^{2}\gamma +b\left(\alpha -\beta \right)\left(5\alpha -4\beta \right)}{5a^{2}}\right\}t+\theta_{0}\right)}\text{,}$$

and

$$q\left(x,t\right)=\mp \frac{4\beta -5\alpha }{4a}\;\csc\;h^{2}\left[\frac{\sqrt{b\left(4\beta -5\alpha \right)}}{4\sqrt{5}a}\;x\right]\;e^{i\left(\left\{\frac{5a^{2}\gamma +b\left(\alpha -\beta \right)\left(5\alpha -4\beta \right)}{5a^{2}}\right\}t+\theta_{0}\right)}\text{,}$$

respectively. These solitons remain valid within

b(4β−5α)>0.

**Result–2**:

$$\lambda_{0}=\lambda_{01}=\lambda_{11}=\lambda_{20}=0,\lambda_{10}=\frac{\eta \left(4\beta -5\alpha \right)}{a},\lambda_{20}=-\eta \lambda_{10},k=\pm \frac{\sqrt{b\left(4\beta -5\alpha \right)}}{2\sqrt{5}a}\text{,}$$

(16) $$\omega =\frac{5a^{2}\gamma +b\left(\alpha -\beta \right)\left(5\alpha -4\beta \right)}{5a^{2}}\text{.}$$

Plugging (16) with the help of (7), (13) paves way to the soliton wave profile

(17) $$q\left(x,t\right)=\frac{\left(4\beta -5\alpha \right)\exp\left[\pm \frac{\sqrt{b\left(4\beta -5\alpha \right)}}{2\sqrt{5}a}\;x\right]}{a{\left(\exp\left[\pm \frac{\sqrt{b\left(4\beta -5\alpha \right)}}{2\sqrt{5}a}\;x\right]+1\right)}^{2}}\;e^{i\left(\left\{\frac{5a^{2}\gamma +b\left(\alpha -\beta \right)\left(5\alpha -4\beta \right)}{5a^{2}}\right\}t+\theta_{0}\right)}\text{.}$$

Setting η=±d in solution (17), we have the quiescent singular and bright solitons

$$q\left(x,t\right)=\mp \frac{4\beta -5\alpha }{4a}\;\csc\;h^{2}\left[\frac{\sqrt{b\left(4\beta -5\alpha \right)}}{4\sqrt{5}a}\;x\right]\;e^{i\left(\left\{\frac{5a^{2}\gamma +b\left(\alpha -\beta \right)\left(5\alpha -4\beta \right)}{5a^{2}}\right\}t+\theta_{0}\right)}\text{,}$$

and

$$q\left(x,t\right)=\pm \frac{4\beta -5\alpha }{4a}\;\sec\;h^{2}\left[\frac{\sqrt{b\left(4\beta -5\alpha \right)}}{4\sqrt{5}a}\;x\right]\;e^{i\left(\left\{\frac{5a^{2}\gamma +b\left(\alpha -\beta \right)\left(5\alpha -4\beta \right)}{5a^{2}}\right\}t+\theta_{0}\right)}\text{,}$$

respectively. The range of applicability for these solitons is

b(4β−5α)>0.

### 3.2 Power law

Eq. (8) turns out to be

$$iq_{t}+a{\left({\left|q\right|}^{n}q\right)}_{xx}+b{\left|q\right|}^{2m}q=\frac{1}{{\left|q\right|}^{2}q^{*}}\left\{\alpha {\left|q\right|}^{2}{\left({\left|q\right|}^{2}\right)}_{xx}-\beta {\left\{{\left({\left|q\right|}^{2}\right)}_{x}\right\}}^{2}\right\}+\gamma q\text{.}$$

Thus, Eq. (10) changes to

(18) $$ak^{2}\left(n+1\right)U^{n+1}U^{\prime \prime }+ak^{2}n\left(n+1\right)U^{n}{U^{\prime }}^{2}+bU^{2m+2}-2\alpha k^{2}UU^{\prime \prime }-2k^{2}\left(\alpha -2\beta \right){U^{\prime }}^{2}+\left(\gamma -\omega \right)U^{2}=0\text{.}$$

We can choose n=m. Hence, Eq. (18) simplifies to

(19) $$ak^{2}\left(m+1\right)U^{m+1}U^{\prime \prime }+ak^{2}m\left(m+1\right)U^{m}{U^{\prime }}^{2}+bU^{2m+2}-2\alpha k^{2}UU^{\prime \prime }-2k^{2}\left(\alpha -2\beta \right){U^{\prime }}^{2}+\left(\gamma -\omega \right)U^{2}=0\text{.}$$

Now, we assume

$$U=V^{\frac{2}{m}}\text{.}$$

In this case, Eq. (19) shapes up as

$$2k^{2}{V^{\prime }}^{2}\left(a\left(m^{2}+3m+2\right)V^{2}+8\beta +2\alpha \left(m-4\right)\right)+2k^{2}mVV^{\prime \prime }\left(a\left(m+1\right)V^{2}-2\alpha \right)+$$

(20) $$m^{2}V^{2}\left(bV^{4}+\gamma -\omega \right)=0\text{.}$$

Eq. (20) yields N=1 by balancing $V^{6}$ with $V^{3}V^{\prime \prime }$, leading us to:

(21) $$V=\lambda_{0}+\lambda_{01}R+\lambda_{10}Q\text{.}$$

Plugging (21) together with (4), (5), (20) leaves us with a polynomial of Q,R and $R^{\prime }$. The polynomial obtained by collecting terms with the same powers of Q, R, and $R^{\prime }$ is set to zero, resulting in a system of equations that can be addressed to derive the outcomes:

**Result–1**:

$$\lambda_{0}=\lambda_{10}=0,\lambda_{01}=\pm \sqrt{\frac{\chi \left(4\beta -\alpha \left(m+4\right)\right)}{a{\left(m+1\right)}^{2}}},k=\sqrt{\frac{bm^{2}\left(4\beta -\alpha \left(m+4\right)\right)}{2a^{2}{\left(m+1\right)}^{3}\left(3m+2\right)}}\text{,}$$

(22) $$\omega =\frac{8b\left(\alpha -\beta \right)\left(\alpha \left(m+4\right)-4\beta \right)}{a^{2}{\left(m+1\right)}^{3}\left(3m+2\right)}+\gamma \text{.}$$

The nonlinear wave profile is acquired by inserting (22) with the help of (6), (21), as indicated below

$$q\left(x,t\right)={\left\{\pm \frac{4c\sqrt{\frac{\chi \left(4\beta -\alpha \left(m+4\right)\right)}{a{\left(m+1\right)}^{2}}}}{4c^{2}\;\exp\left[\sqrt{\frac{bm^{2}\left(4\beta -\alpha \left(m+4\right)\right)}{2a^{2}{\left(m+1\right)}^{3}\left(3m+2\right)}}\;x\right]+\chi \;\exp\left[-\sqrt{\frac{bm^{2}\left(4\beta -\alpha \left(m+4\right)\right)}{2a^{2}{\left(m+1\right)}^{3}\left(3m+2\right)}}\;x\right]}\right\}}^{\frac{2}{m}}$$

(23) $$\times e^{i\left(\left\{\frac{8b\left(\alpha -\beta \right)\left(\alpha \left(m+4\right)-4\beta \right)}{a^{2}{\left(m+1\right)}^{3}\left(3m+2\right)}+\gamma \right\}t+\theta_{0}\right)}\text{.}$$

Taking $\chi =\pm 4c^{2}$ in solution (23), we have the quiescent bright and singular solitons

$$q\left(x,t\right)={\left\{\pm \sqrt{\frac{4\beta -\alpha \left(m+4\right)}{a{\left(m+1\right)}^{2}}}\mathrm{sech}\left[\sqrt{\frac{bm^{2}\left(4\beta -\alpha \left(m+4\right)\right)}{2a^{2}{\left(m+1\right)}^{3}\left(3m+2\right)}}\;x\right]\right\}}^{\frac{2}{m}}$$

(24) $$\times e^{i\left(\left\{\frac{8b\left(\alpha -\beta \right)\left(\alpha \left(m+4\right)-4\beta \right)}{a^{2}{\left(m+1\right)}^{3}\left(3m+2\right)}+\gamma \right\}t+\theta_{0}\right)}\text{,}$$

and

$$q\left(x,t\right)={\left\{\pm \sqrt{-\frac{4\beta -\alpha \left(m+4\right)}{a{\left(m+1\right)}^{2}}}\mathrm{csch}\left[\sqrt{\frac{bm^{2}\left(4\beta -\alpha \left(m+4\right)\right)}{2a^{2}{\left(m+1\right)}^{3}\left(3m+2\right)}}\;x\right]\right\}}^{\frac{2}{m}}$$

$$\times e^{i\left(\left\{\frac{8b\left(\alpha -\beta \right)\left(\alpha \left(m+4\right)-4\beta \right)}{a^{2}{\left(m+1\right)}^{3}\left(3m+2\right)}+\gamma \right\}t+\theta_{0}\right)}\text{,}$$

respectively. These solitons are valid for

b(4β−α(m+4))>0.

**Fig. 1:**
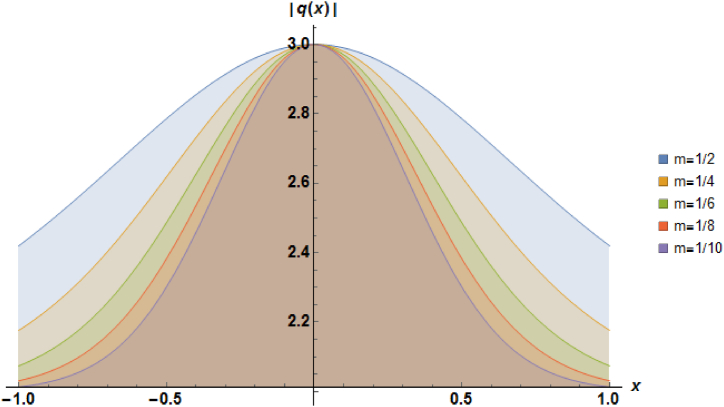
Characteristics of a stationary bright soliton profile.

Plots of the soliton described by equation (24) with b=1, β=1, α=−1 and a=1 are illustrated in Fig. 1.

**Result–2**:

$$\lambda_{0}=\pm \sqrt{-\frac{\alpha m}{a{\left(m+1\right)}^{2}}},\lambda_{10}=\mp 2\eta \lambda_{0},k=\sqrt{\frac{2\alpha bm^{3}}{a^{2}{\left(m+1\right)}^{3}\left(3m+2\right)}}\text{,}$$

(25) $$\omega =\frac{a^{2}\gamma {\left(m+1\right)}^{4}+\alpha^{2}bm^{2}}{a^{2}{\left(m+1\right)}^{4}},\beta =\alpha \left(1-\frac{m}{4}\right)\text{.}$$

Putting (25) with the aid of (7), (21) provides us the nonlinear waveform

$$q\left(x,t\right)={\left\{\pm \sqrt{-\frac{\alpha m}{a{\left(m+1\right)}^{2}}}\left(1-\frac{2\eta }{d\;\exp\left[\sqrt{\frac{2\alpha bm^{3}}{a^{2}{\left(m+1\right)}^{3}\left(3m+2\right)}}\;x\right]+\eta }\right)\right\}}^{\frac{2}{m}}$$

(26) $$\times e^{i\left(\left\{\frac{a^{2}\gamma {\left(m+1\right)}^{4}+\alpha^{2}bm^{2}}{a^{2}{\left(m+1\right)}^{4}}\right\}t+\theta_{0}\right)}\text{.}$$

Assuming η=±d in solution (26) leads to the singular and dark solitons

$$q\left(x,t\right)={\left\{\mp \sqrt{-\frac{\alpha m}{a{\left(m+1\right)}^{2}}}\coth\left[\sqrt{\frac{\alpha bm^{3}}{2a^{2}{\left(m+1\right)}^{3}\left(3m+2\right)}}\;x\right]\right\}}^{\frac{2}{m}}\;e^{i\left(\left\{\frac{a^{2}\gamma {\left(m+1\right)}^{4}+\alpha^{2}bm^{2}}{a^{2}{\left(m+1\right)}^{4}}\right\}t+\theta_{0}\right)}\text{,}$$

and

(27) $$q\left(x,t\right)={\left\{\pm \sqrt{-\frac{\alpha m}{a{\left(m+1\right)}^{2}}}\tanh\left[\sqrt{\frac{\alpha bm^{3}}{2a^{2}{\left(m+1\right)}^{3}\left(3m+2\right)}}\;x\right]\right\}}^{\frac{2}{m}}\;e^{i\left(\left\{\frac{a^{2}\gamma {\left(m+1\right)}^{4}+\alpha^{2}bm^{2}}{a^{2}{\left(m+1\right)}^{4}}\right\}t+\theta_{0}\right)}\text{,}$$

respectively. These solitons are valid for

bα>0.

**Fig. 2:**
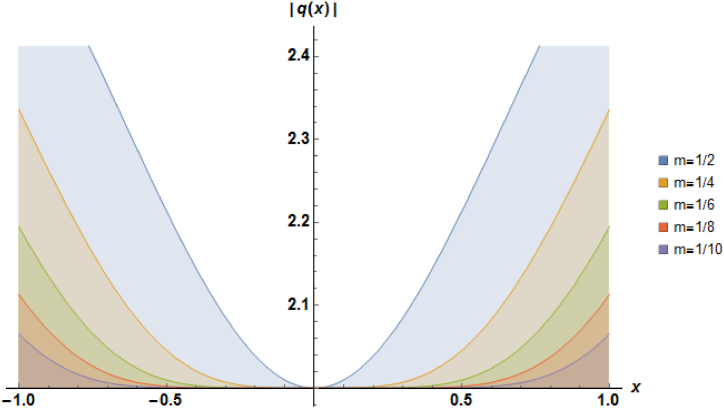
Characteristics of a stationary dark soliton profile.

Plots of the soliton described by equation (27) with b=1, α=1 and a=−1 are demonstrated in Fig. 2.

### 3.3 Parabolic law

Eq. (8) sticks out as

$$iq_{t}+a{\left({\left|q\right|}^{n}q\right)}_{xx}+\left(b_{1}{\left|q\right|}^{2}+b_{2}{\left|q\right|}^{4}\right)q=\frac{1}{{\left|q\right|}^{2}q^{*}}\left\{\alpha {\left|q\right|}^{2}{\left({\left|q\right|}^{2}\right)}_{xx}-\beta {\left\{{\left({\left|q\right|}^{2}\right)}_{x}\right\}}^{2}\right\}+\gamma q\text{.}$$

Thus, the expression for Eq. (10) can be derived as follows

(28) $$ak^{2}\left(n+1\right)U^{n+1}U^{\prime \prime }+ak^{2}n\left(n+1\right)U^{n}{U^{\prime }}^{2}+b_{1}U^{4}+b_{2}U^{6}-2\alpha k^{2}UU^{\prime \prime }-2k^{2}\left(\alpha -2\beta \right){U^{\prime }}^{2}+\left(\gamma -\omega \right)U^{2}=0\text{.}$$

For integrability, we take n=2. Therefore, Eq. (28) translates to

(29) $$3ak^{2}U^{3}U^{\prime \prime }+6ak^{2}U^{2}{U^{\prime }}^{2}+b_{1}U^{4}+b_{2}U^{6}-2\alpha k^{2}UU^{\prime \prime }-2k^{2}\left(\alpha -2\beta \right){U^{\prime }}^{2}+\left(\gamma -\omega \right)U^{2}=0\text{.}$$

Balancing $U^{3}U^{\prime \prime }$ with $U^{6}$ in Eq. (29) gives rise to N=1. As a result, one secures

(30) $$U=\lambda_{0}+\lambda_{01}R+\lambda_{10}Q\text{.}$$

Putting (30) along with (4), (5), (29) allows us a polynomial of Q,R and $R^{\prime }$. Upon setting all terms with the same powers to zero, one retrieves a system of equations that can be addressed to secure the findings:

**Result–1**:

$$\lambda_{0}=\lambda_{10}=0,\lambda_{01}=\pm \sqrt{-\frac{2\chi \left(6ab_{1}+b_{2}\left(3\alpha -2\beta \right)\right)}{9ab_{2}}},k=\frac{\sqrt{b_{2}\left(2\beta -3\alpha \right)-6ab_{1}}}{3\sqrt{6}a}\text{,}$$

(31) $$\omega =\frac{2b_{2}\left(3\alpha^{2}-5\alpha \beta +2\beta^{2}\right)}{27a^{2}}+\frac{4b_{1}\left(\alpha -\beta \right)}{9a}+\gamma \text{.}$$

Inserting (31) with the help of (6), (30) allows us the soliton wave profile

$$q\left(x,t\right)=\pm \frac{4c\sqrt{-\frac{2\chi \left(6ab_{1}+b_{2}\left(3\alpha -2\beta \right)\right)}{9ab_{2}}}}{4c^{2}\;\exp\left[\frac{\sqrt{b_{2}\left(2\beta -3\alpha \right)-6ab_{1}}}{3\sqrt{6}a}\;x\right]+\chi \;\exp\left[-\frac{\sqrt{b_{2}\left(2\beta -3\alpha \right)-6ab_{1}}}{3\sqrt{6}a}\;x\right]}$$

(32) $$\times e^{i\left(\left\{\frac{2b_{2}\left(3\alpha^{2}-5\alpha \beta +2\beta^{2}\right)}{27a^{2}}+\frac{4b_{1}\left(\alpha -\beta \right)}{9a}+\gamma \right\}t+\theta_{0}\right)}\text{.}$$

Choosing $\chi =\pm 4c^{2}$ in solution (32) leaves us with the quiescent singular and bright solitons

$$q\left(x,t\right)=\pm \sqrt{\frac{2\left(6ab_{1}+b_{2}\left(3\alpha -2\beta \right)\right)}{9ab_{2}}}\mathrm{csch}\left[\frac{\sqrt{b_{2}\left(2\beta -3\alpha \right)-6ab_{1}}}{3\sqrt{6}a}\;x\right]$$

$$\times e^{i\left(\left\{\frac{2b_{2}\left(3\alpha^{2}-5\alpha \beta +2\beta^{2}\right)}{27a^{2}}+\frac{4b_{1}\left(\alpha -\beta \right)}{9a}+\gamma \right\}t+\theta_{0}\right)}\text{,}$$

and

$$q\left(x,t\right)=\pm \sqrt{-\frac{2\left(6ab_{1}+b_{2}\left(3\alpha -2\beta \right)\right)}{9ab_{2}}}\mathrm{sech}\left[\frac{\sqrt{b_{2}\left(2\beta -3\alpha \right)-6ab_{1}}}{3\sqrt{6}a}\;x\right]$$

$$\times e^{i\left(\left\{\frac{2b_{2}\left(3\alpha^{2}-5\alpha \beta +2\beta^{2}\right)}{27a^{2}}+\frac{4b_{1}\left(\alpha -\beta \right)}{9a}+\gamma \right\}t+\theta_{0}\right)}\text{,}$$

respectively. These solitons are valid for

$$b_{2}\left(2\beta -3\alpha \right)-6ab_{1}>0\text{.}$$

**Result–2**:

$$\lambda_{0}=\mp \sqrt{-\frac{6ab_{1}+2\alpha b_{2}}{9ab_{2}}},\lambda_{01}=0,\lambda_{10}=-2\eta \lambda_{0},k=\frac{\sqrt{6ab_{1}+2\alpha b_{2}}}{3\sqrt{3}a}\text{,}$$

(33) $$\omega =\frac{4\alpha^{2}b_{2}}{81a^{2}}+\frac{2\alpha b_{1}}{27a}-\frac{2b_{1}^{2}}{9b_{2}}+\gamma ,\beta =\frac{\alpha }{2}\text{.}$$

Plugging (33) with the aid of (7), (30) gives way to the nonlinear waveform

(34) $$q\left(x,t\right)=\mp \sqrt{-\frac{6ab_{1}+2\alpha b_{2}}{9ab_{2}}}\left(1-\frac{2\eta }{d\;\exp\left[\frac{\sqrt{6ab_{1}+2\alpha b_{2}}}{3\sqrt{3}a}\;x\right]+\eta }\right)e^{i\left(\left\{\frac{4\alpha^{2}b_{2}}{81a^{2}}+\frac{2\alpha b_{1}}{27a}-\frac{2b_{1}^{2}}{9b_{2}}+\gamma \right\}t+\theta_{0}\right)}\text{.}$$

The singular and dark solitons can be obtained by assuming η=±d in solution (34), as given below

$$q\left(x,t\right)=\pm \sqrt{-\frac{6ab_{1}+2\alpha b_{2}}{9ab_{2}}}\coth\left[\frac{\sqrt{6ab_{1}+2\alpha b_{2}}}{6\sqrt{3}a}\;x\right]\;e^{i\left(\left\{\frac{4\alpha^{2}b_{2}}{81a^{2}}+\frac{2\alpha b_{1}}{27a}-\frac{2b_{1}^{2}}{9b_{2}}+\gamma \right\}t+\theta_{0}\right)}\text{,}$$

and

$$q\left(x,t\right)=\mp \sqrt{-\frac{6ab_{1}+2\alpha b_{2}}{9ab_{2}}}\tanh\left[\frac{\sqrt{6ab_{1}+2\alpha b_{2}}}{6\sqrt{3}a}\;x\right]\;e^{i\left(\left\{\frac{4\alpha^{2}b_{2}}{81a^{2}}+\frac{2\alpha b_{1}}{27a}-\frac{2b_{1}^{2}}{9b_{2}}+\gamma \right\}t+\theta_{0}\right)}\text{,}$$

respectively. These solitons are valid for

$$6ab_{1}+2\alpha b_{2}>0\text{.}$$

### 3.4 Dual–power law

Eq. (8) sticks out as

$$iq_{t}+a{\left({\left|q\right|}^{n}q\right)}_{xx}+\left(b_{1}{\left|q\right|}^{2m}+b_{2}{\left|q\right|}^{4m}\right)q=\frac{1}{{\left|q\right|}^{2}q^{*}}\left\{\alpha {\left|q\right|}^{2}{\left({\left|q\right|}^{2}\right)}_{xx}-\beta {\left\{{\left({\left|q\right|}^{2}\right)}_{x}\right\}}^{2}\right\}+\gamma q\text{.}$$

Therefore, Eq. (10) reads as

$$ak^{2}\left(n+1\right)U^{n+1}U^{\prime \prime }+ak^{2}n\left(n+1\right)U^{n}{U^{\prime }}^{2}+b_{1}U^{2m+2}+b_{2}U^{4m+2}-2\alpha k^{2}UU^{\prime \prime }-$$

(35) $$2k^{2}\left(\alpha -2\beta \right){U^{\prime }}^{2}+\left(\gamma -\omega \right)U^{2}=0\text{.}$$

We can choose n=2m. As a result, Eq. (35) turns out to be

$$ak^{2}\left(2m+1\right)U^{2m+1}U^{\prime \prime }+ak^{2}2m\left(2m+1\right)U^{2m}{U^{\prime }}^{2}+b_{1}U^{2m+2}+b_{2}U^{4m+2}-2\alpha k^{2}UU^{\prime \prime }-$$

(36) $$2k^{2}\left(\alpha -2\beta \right){U^{\prime }}^{2}+\left(\gamma -\omega \right)U^{2}=0\text{.}$$

Now, we assume

$$U=V^{\frac{1}{m}}\text{.}$$

As a result, Eq. (36) appears as

$$k^{2}{V^{\prime }}^{2}\left(a\left(2m^{2}+3m+1\right)V^{2}+4\beta +2\alpha \left(m-2\right)\right)+k^{2}mVV^{\prime \prime }\left(a\left(2m+1\right)V^{2}-2\alpha \right)+$$

(37) $$m^{2}V^{2}\left(b_{2}V^{4}+b_{1}V^{2}+\gamma -\omega \right)=0\text{.}$$

One can determine the constant N in Eq. (37) by equating $V^{6}$ with $V^{3}V^{\prime \prime }$, which yields N=1 and the following expression:

(38) $$V=\lambda_{0}+\lambda_{01}R+\lambda_{10}Q\text{.}$$

Substituting (38) along with (4), (5), (37) causes to a polynomial of Q,R and $R^{\prime }$. Collecting terms with the same powers and setting them to zero results in a system of equations, which can be addressed to secure the outcomes:

**Result–1**:

$$\lambda_{0}=\lambda_{10}=0,\lambda_{01}=\pm \sqrt{-\frac{\chi \left(ab_{1}\left(6m^{2}+5m+1\right)+2b_{2}\left(\alpha \left(m+2\right)-2\beta \right)\right)}{ab_{2}{\left(2m+1\right)}^{2}}}\text{,}$$

$$k=m\sqrt{-\frac{ab_{1}\left(6m^{2}+5m+1\right)+2b_{2}\left(\alpha \left(m+2\right)-2\beta \right)}{a^{2}{\left(2m+1\right)}^{3}\left(3m+1\right)}}\text{,}$$

(39) $$\omega =\frac{a^{2}\gamma \left(3m+1\right){\left(2m+1\right)}^{3}+4ab_{1}\left(6m^{2}+5m+1\right)\left(\alpha -\beta \right)+8b_{2}\left(\alpha -\beta \right)\left(\alpha \left(m+2\right)-2\beta \right)}{a^{2}{\left(2m+1\right)}^{3}\left(3m+1\right)}\text{.}$$

Putting (39) along with (6), (38) yields the optoelectronic wave field

$$q\left(x,t\right)={\left\{\begin{array}{c} \pm \sqrt{-\frac{\chi \left(ab_{1}\left(6m^{2}+5m+1\right)+2b_{2}\left(\alpha \left(m+2\right)-2\beta \right)\right)}{ab_{2}{\left(2m+1\right)}^{2}}}\\ \times \frac{4c}{\left(\begin{array}{c} 4c^{2}\;exp\left[m\sqrt{-\frac{ab_{1}\left(6m^{2}+5m+1\right)+2b_{2}\left(\alpha \left(m+2\right)-2\beta \right)}{a^{2}{\left(2m+1\right)}^{3}\left(3m+1\right)}}\;x\right]\\ +\chi \;exp\left[-m\sqrt{-\frac{ab_{1}\left(6m^{2}+5m+1\right)+2b_{2}\left(\alpha \left(m+2\right)-2\beta \right)}{a^{2}{\left(2m+1\right)}^{3}\left(3m+1\right)}}\;x\right] \end{array}\right)} \end{array}\right\}}^{\frac{1}{m}}$$

(40) $$\times e^{i\left(\left\{\frac{a^{2}\gamma \left(3m+1\right){\left(2m+1\right)}^{3}+4ab_{1}\left(6m^{2}+5m+1\right)\left(\alpha -\beta \right)+8b_{2}\left(\alpha -\beta \right)\left(\alpha \left(m+2\right)-2\beta \right)}{a^{2}{\left(2m+1\right)}^{3}\left(3m+1\right)}\right\}t+\theta_{0}\right)}\text{.}$$

When $\chi =\pm 4c^{2}$ in solution (40), we arrive at the quiescent singular and bright solitons

$$q\left(x,t\right)={\left\{\pm \sqrt{\frac{ab_{1}\left(6m^{2}+5m+1\right)+2b_{2}\left(\alpha \left(m+2\right)-2\beta \right)}{ab_{2}{\left(2m+1\right)}^{2}}}\;\mathrm{csch}\left[m\sqrt{-\frac{ab_{1}\left(6m^{2}+5m+1\right)+2b_{2}\left(\alpha \left(m+2\right)-2\beta \right)}{a^{2}{\left(2m+1\right)}^{3}\left(3m+1\right)}}\;x\right]\right\}}^{\frac{1}{m}}$$

$$\times e^{i\left(\left\{\frac{a^{2}\gamma \left(3m+1\right){\left(2m+1\right)}^{3}+4ab_{1}\left(6m^{2}+5m+1\right)\left(\alpha -\beta \right)+8b_{2}\left(\alpha -\beta \right)\left(\alpha \left(m+2\right)-2\beta \right)}{a^{2}{\left(2m+1\right)}^{3}\left(3m+1\right)}\right\}t+\theta_{0}\right)}\text{,}$$

and

$$q\left(x,t\right)={\left\{\pm \sqrt{-\frac{ab_{1}\left(6m^{2}+5m+1\right)+2b_{2}\left(\alpha \left(m+2\right)-2\beta \right)}{ab_{2}{\left(2m+1\right)}^{2}}}\mathrm{sech}\left[m\sqrt{-\frac{ab_{1}\left(6m^{2}+5m+1\right)+2b_{2}\left(\alpha \left(m+2\right)-2\beta \right)}{a^{2}{\left(2m+1\right)}^{3}\left(3m+1\right)}}\;x\right]\right\}}^{\frac{1}{m}}$$

(41) $$\times e^{i\left(\left\{\frac{a^{2}\gamma \left(3m+1\right){\left(2m+1\right)}^{3}+4ab_{1}\left(6m^{2}+5m+1\right)\left(\alpha -\beta \right)+8b_{2}\left(\alpha -\beta \right)\left(\alpha \left(m+2\right)-2\beta \right)}{a^{2}{\left(2m+1\right)}^{3}\left(3m+1\right)}\right\}t+\theta_{0}\right)}\text{,}$$

respectively. These solitons are valid for

$$ab_{1}\left(6m^{2}+5m+1\right)+2b_{2}\left(\alpha \left(m+2\right)-2\beta \right)<0\text{.}$$

**Fig. 3:**
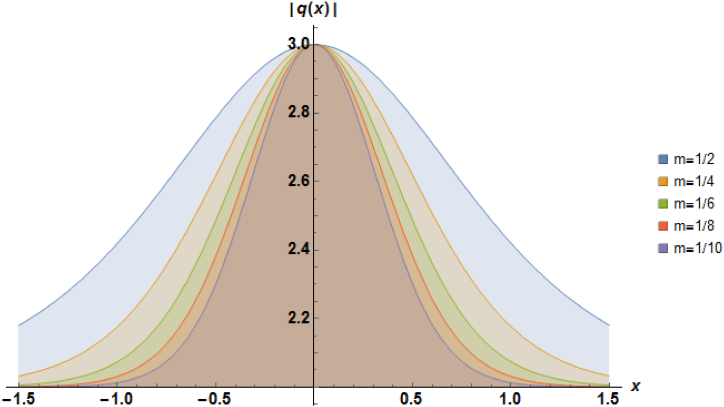
Characteristics of a stationary bright soliton profile.

Plots of the soliton described by equation (41) with β=−1, α=1, $b_{2}=-1$, a=1 and $b_{1}=-1$ are depicted in Fig. 3.

**Result–2**:

$$\lambda_{01}=0,\lambda_{0}=\pm \sqrt{\frac{-ab_{1}\left(6m^{2}+5m+1\right)-4\alpha b_{2}m}{2ab_{2}{\left(2m+1\right)}^{2}}},\lambda_{10}=-2\eta \lambda_{0},k=\sqrt{\frac{2m^{2}\left(ab_{1}\left(6m^{2}+5m+1\right)+4\alpha b_{2}m\right)}{a^{2}{\left(2m+1\right)}^{3}\left(3m+1\right)}}\text{,}$$

$$\omega =\frac{4b_{2}\left(a^{2}\gamma {\left(2m+1\right)}^{4}+4\alpha^{2}b_{2}m^{2}\right)+8a\alpha b_{1}b_{2}\left(2m+1\right)m^{2}-b_{1}^{2}\left(3m^{2}+4m+1\right){\left(2am+a\right)}^{2}}{4a^{2}b_{2}{\left(2m+1\right)}^{4}}\text{,}$$

(42) $$\beta =\alpha \left(1-\frac{m}{2}\right)\text{.}$$

Inserting (42) with the usage of (7), (38) gives rise to the nonlinear wave profile

$$q\left(x,t\right)={\left\{\pm \sqrt{\frac{-ab_{1}\left(6m^{2}+5m+1\right)-4\alpha b_{2}m}{2ab_{2}{\left(2m+1\right)}^{2}}}\left(1-\frac{2\eta }{d\;\exp\left[\sqrt{\frac{2m^{2}\left(ab_{1}\left(6m^{2}+5m+1\right)+4\alpha b_{2}m\right)}{a^{2}{\left(2m+1\right)}^{3}\left(3m+1\right)}}\;x\right]+\eta }\right)\right\}}^{\frac{1}{m}}$$

(43) $$\times e^{i\left(\left\{\frac{4b_{2}\left(a^{2}\gamma {\left(2m+1\right)}^{4}+4\alpha^{2}b_{2}m^{2}\right)+8a\alpha b_{1}b_{2}\left(2m+1\right)m^{2}-b_{1}^{2}\left(3m^{2}+4m+1\right){\left(2am+a\right)}^{2}}{4a^{2}b_{2}{\left(2m+1\right)}^{4}}\right\}t+\theta_{0}\right)}\text{.}$$

Solution (43) yields the quiescent singular and dark solitons if we set η=±d, as indicated below

$$q\left(x,t\right)={\left\{\mp \sqrt{\frac{-ab_{1}\left(6m^{2}+5m+1\right)-4\alpha b_{2}m}{2ab_{2}{\left(2m+1\right)}^{2}}}\coth\left[\sqrt{\frac{m^{2}\left(ab_{1}\left(6m^{2}+5m+1\right)+4\alpha b_{2}m\right)}{2a^{2}{\left(2m+1\right)}^{3}\left(3m+1\right)}}\;x\right]\right\}}^{\frac{1}{m}}$$

$$\times e^{i\left(\left\{\frac{4b_{2}\left(a^{2}\gamma {\left(2m+1\right)}^{4}+4\alpha^{2}b_{2}m^{2}\right)+8a\alpha b_{1}b_{2}\left(2m+1\right)m^{2}-b_{1}^{2}\left(3m^{2}+4m+1\right){\left(2am+a\right)}^{2}}{4a^{2}b_{2}{\left(2m+1\right)}^{4}}\right\}t+\theta_{0}\right)}\text{,}$$

and

$$q\left(x,t\right)={\left\{\pm \sqrt{\frac{-ab_{1}\left(6m^{2}+5m+1\right)-4\alpha b_{2}m}{2ab_{2}{\left(2m+1\right)}^{2}}}\tanh\left[\sqrt{\frac{m^{2}\left(ab_{1}\left(6m^{2}+5m+1\right)+4\alpha b_{2}m\right)}{2a^{2}{\left(2m+1\right)}^{3}\left(3m+1\right)}}\;x\right]\right\}}^{\frac{1}{m}}$$

(44) $$\times e^{i\left(\left\{\frac{4b_{2}\left(a^{2}\gamma {\left(2m+1\right)}^{4}+4\alpha^{2}b_{2}m^{2}\right)+8a\alpha b_{1}b_{2}\left(2m+1\right)m^{2}-b_{1}^{2}\left(3m^{2}+4m+1\right){\left(2am+a\right)}^{2}}{4a^{2}b_{2}{\left(2m+1\right)}^{4}}\right\}t+\theta_{0}\right)}\text{,}$$

respectively. These solitons are valid for

$$ab_{1}\left(6m^{2}+5m+1\right)+4\alpha b_{2}m>0\text{.}$$

**Fig. 4:**
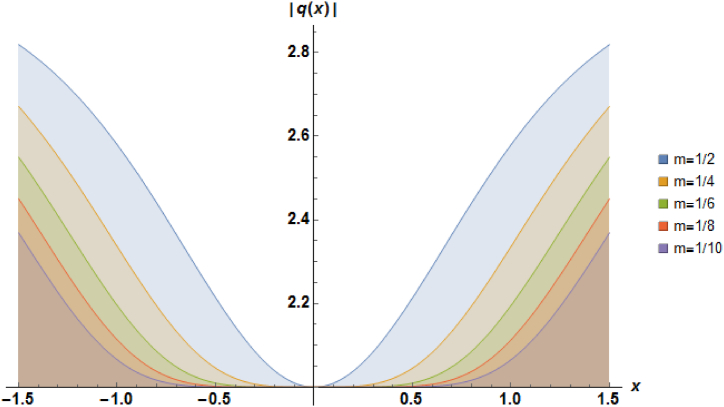
Characteristics of a stationary dark soliton profile.

Plots of the soliton described by equation (44) with a=1, $b_{1}=1$, α=1 and $b_{2}=1$ are presented in Fig. 4.

### 3.5 Quadratic–cubic law

Eq. (8) appears as

$$iq_{t}+a{\left({\left|q\right|}^{n}q\right)}_{xx}+\left(b_{1}\left|q\right|+b_{2}{\left|q\right|}^{2}\right)q=\frac{1}{{\left|q\right|}^{2}q^{*}}\left\{\alpha {\left|q\right|}^{2}{\left({\left|q\right|}^{2}\right)}_{xx}-\beta {\left\{{\left({\left|q\right|}^{2}\right)}_{x}\right\}}^{2}\right\}+\gamma q\text{.}$$

We can deduce the evolution of Eq. (10) by using the given expressions as follows

(45) $$ak^{2}\left(n+1\right)U^{n+1}U^{\prime \prime }+ak^{2}n\left(n+1\right)U^{n}{U^{\prime }}^{2}+b_{1}U^{3}+b_{2}U^{4}-2\alpha k^{2}UU^{\prime \prime }-2k^{2}\left(\alpha -2\beta \right){U^{\prime }}^{2}+\left(\gamma -\omega \right)U^{2}=0\text{.}$$

For integrability, we set n=1. So Eq. (45) stands as

(46) $$2ak^{2}U^{2}U^{\prime \prime }+2ak^{2}U{U^{\prime }}^{2}+b_{1}U^{3}+b_{2}U^{4}-2\alpha k^{2}UU^{\prime \prime }-2k^{2}\left(\alpha -2\beta \right){U^{\prime }}^{2}+\left(\gamma -\omega \right)U^{2}=0\text{.}$$

Balancing $U^{4}$ with $UU^{\prime \prime }$ in Eq. (46) gives N=1. As a result, we arrive at

(47) $$U=\lambda_{0}+\lambda_{01}R+\lambda_{10}Q\text{.}$$

Inserting (47) along with (4), (5), (46) allows us a polynomial of Q,R and $R^{\prime }$. Equating all terms with the same powers to zero provides us a system of equations, the solution of which gives us the following outcomes:

**Result–1**:

$$\lambda_{0}=\lambda_{01}=\lambda_{10}=\lambda_{11}=\lambda_{20}=0,\lambda_{02}=\frac{1}{4}\chi \left(\frac{4\beta -5\alpha }{a}-\frac{5b_{1}}{b_{2}}\right),k=\pm \frac{\sqrt{b_{2}\left(4\beta -5\alpha \right)-5ab_{1}}}{4\sqrt{5}a}\text{,}$$

(48) $$\omega =\frac{b_{2}\left(5\alpha^{2}-9\alpha \beta +4\beta^{2}\right)}{5a^{2}}+\frac{b_{1}\left(\alpha -\beta \right)}{a}+\gamma \text{.}$$

The soliton wave profile emerges from the substitution of (48) with (6), (47), as presented below

$$q\left(x,t\right)=\frac{1}{4}\chi \left(\frac{4\beta -5\alpha }{a}-\frac{5b_{1}}{b_{2}}\right){\left(\frac{4c}{4c^{2}\;\exp\left[\pm \frac{\sqrt{b_{2}\left(4\beta -5\alpha \right)-5ab_{1}}}{4\sqrt{5}a}\;x\right]+\chi \;\exp\left[\mp \frac{\sqrt{b_{2}\left(4\beta -5\alpha \right)-5ab_{1}}}{4\sqrt{5}a}\;x\right]}\right)}^{2}$$

(49) $$\times e^{i\left(\left\{\frac{b_{2}\left(5\alpha^{2}-9\alpha \beta +4\beta^{2}\right)}{5a^{2}}+\frac{b_{1}\left(\alpha -\beta \right)}{a}+\gamma \right\}t+\theta_{0}\right)}\text{.}$$

The solution given in (49) yields the quiescent bright and singular solitons when χ is set to $\pm 4c^{2}$, as recovered below

$$q\left(x,t\right)=\frac{1}{4}\left(\frac{4\beta -5\alpha }{a}-\frac{5b_{1}}{b_{2}}\right)\sec\;h^{2}\left[\frac{\sqrt{b_{2}\left(4\beta -5\alpha \right)-5ab_{1}}}{4\sqrt{5}a}\;x\right]$$

$$\times e^{i\left(\left\{\frac{b_{2}\left(5\alpha^{2}-9\alpha \beta +4\beta^{2}\right)}{5a^{2}}+\frac{b_{1}\left(\alpha -\beta \right)}{a}+\gamma \right\}t+\theta_{0}\right)}\text{,}$$

and

$$q\left(x,t\right)=\frac{1}{4}\left(\frac{5\alpha -4\beta }{a}+\frac{5b_{1}}{b_{2}}\right)\csc\;h^{2}\left[\frac{\sqrt{b_{2}\left(4\beta -5\alpha \right)-5ab_{1}}}{4\sqrt{5}a}\;x\right]$$

$$\times e^{i\left(\left\{\frac{b_{2}\left(5\alpha^{2}-9\alpha \beta +4\beta^{2}\right)}{5a^{2}}+\frac{b_{1}\left(\alpha -\beta \right)}{a}+\gamma \right\}t+\theta_{0}\right)}\text{,}$$

respectively. These solitons are valid for

$$b_{2}\left(4\beta -5\alpha \right)-5ab_{1}>0\text{.}$$

**Result–2**:

$$\lambda_{0}=\lambda_{01}=\lambda_{11}=\lambda_{02}=\lambda_{10}=0,\lambda_{10}=\eta \left(\frac{4\beta -5\alpha }{a}-\frac{5b_{1}}{b_{2}}\right),\lambda_{20}=-\eta \lambda_{10},k=\pm \frac{\sqrt{b_{2}\left(4\beta -5\alpha \right)-5ab_{1}}}{2\sqrt{5}a}\text{,}$$

(50) $$\omega =\frac{b_{2}\left(5\alpha^{2}-9\alpha \beta +4\beta^{2}\right)}{5a^{2}}+\frac{b_{1}\left(\alpha -\beta \right)}{a}+\gamma \text{.}$$

Putting (50) together with (7), (47) leads to the nonlinear waveform

$$q\left(x,t\right)=\eta \left(\frac{4\beta -5\alpha }{a}-\frac{5b_{1}}{b_{2}}\right)\frac{d\;\exp\left[\pm \frac{\sqrt{b_{2}\left(4\beta -5\alpha \right)-5ab_{1}}}{2\sqrt{5}a}\;x\right]}{{\left(d\;\exp\left[\pm \frac{\sqrt{b_{2}\left(4\beta -5\alpha \right)-5ab_{1}}}{2\sqrt{5}a}\;x\right]+\eta \right)}^{2}}$$

(51) $$\times e^{i\left(\left\{\frac{b_{2}\left(5\alpha^{2}-9\alpha \beta +4\beta^{2}\right)}{5a^{2}}+\frac{b_{1}\left(\alpha -\beta \right)}{a}+\gamma \right\}t+\theta_{0}\right)}\text{.}$$

The quiescent singular and bright solitons can be obtained from solution (51) by setting η=±d, as extracted below

$$q\left(x,t\right)=\frac{1}{4}\left(\frac{5\alpha -4\beta }{a}+\frac{5b_{1}}{b_{2}}\right)\csc\;h^{2}\left[\frac{\sqrt{b_{2}\left(4\beta -5\alpha \right)-5ab_{1}}}{4\sqrt{5}a}\;x\right]$$

$$\times e^{i\left(\left\{\frac{b_{2}\left(5\alpha^{2}-9\alpha \beta +4\beta^{2}\right)}{5a^{2}}+\frac{b_{1}\left(\alpha -\beta \right)}{a}+\gamma \right\}t+\theta_{0}\right)}\text{,}$$

and

$$q\left(x,t\right)=\frac{1}{4}\left(\frac{4\beta -5\alpha }{a}-\frac{5b_{1}}{b_{2}}\right)\sec\;h^{2}\left[\frac{\sqrt{b_{2}\left(4\beta -5\alpha \right)-5ab_{1}}}{4\sqrt{5}a}\;x\right]$$

$$\times e^{i\left(\left\{\frac{b_{2}\left(5\alpha^{2}-9\alpha \beta +4\beta^{2}\right)}{5a^{2}}+\frac{b_{1}\left(\alpha -\beta \right)}{a}+\gamma \right\}t+\theta_{0}\right)}\text{,}$$

respectively. These solitons are valid for

$$b_{2}\left(4\beta -5\alpha \right)-5ab_{1}>0\text{.}$$

### 3.6 Generalized quadratic–cubic law

Eq. (8) collapses to

$$iq_{t}+a{\left({\left|q\right|}^{n}q\right)}_{xx}+\left(b_{1}{\left|q\right|}^{m}+b_{2}{\left|q\right|}^{2m}\right)q=\frac{1}{{\left|q\right|}^{2}q^{*}}\left\{\alpha {\left|q\right|}^{2}{\left({\left|q\right|}^{2}\right)}_{xx}-\beta {\left\{{\left({\left|q\right|}^{2}\right)}_{x}\right\}}^{2}\right\}+\gamma q\text{.}$$

Therefore, the resulting expression for equation (10) is:

(52) $$ak^{2}\left(n+1\right)U^{n+1}U^{\prime \prime }+ak^{2}n\left(n+1\right)U^{n}{U^{\prime }}^{2}+b_{1}U^{m+2}+b_{2}U^{2m+2}-2\alpha k^{2}UU^{\prime \prime }-2k^{2}\left(\alpha -2\beta \right){U^{\prime }}^{2}+\left(\gamma -\omega \right)U^{2}=0\text{.}$$

We can choose n=m. So Eq. (52) changes to

$$ak^{2}\left(m+1\right)U^{m+1}U^{\prime \prime 2}m\left(m+1\right)U^{m}U^{\prime 2}+b_{1}U^{m+2}+b_{2}U^{2m+2}-2\alpha k^{2}UU^{\prime \prime 2}\left(\alpha -2\beta \right)U^{\prime 2}+$$

(53) $$\left(\gamma -\omega \right)U^{2}=0\text{.}$$

Now, we assume

$$U=V^{\frac{2}{m}}\text{.}$$

Thus, Eq. (53) takes the form

$$2k^{2}V^{\prime 2}\left(a\left(m^{2}+3m+2\right)V^{2}+8\beta +2\alpha \left(m-4\right)\right)+2k^{2}mVV^{\prime \prime }\left(a\left(m+1\right)V^{2}-2\alpha \right)+$$

(54) $$m^{2}V^{2}\left(b_{2}V^{4}+b_{1}V^{2}+\gamma -\omega \right)=0\text{.}$$

We can obtain N=1 by balancing the terms $V^{6}$ and $V^{3}V^{\prime \prime }$ in equation (54). As a result, we arrive at the following outcome:

(55) $$V=\lambda_{0}+\lambda_{01}R+\lambda_{10}Q\text{.}$$

Plugging equation (55) along with (4), (5) into equation (54) produces a polynomial involving Q, R, and $R^{\prime }$. We can determine the solutions by collecting all terms with the same powers, setting them to zero, and solving the resulting system of equations, which secures the findings:

**Result–1**:

$$\lambda_{0}=\lambda_{10}=0,\lambda_{01}=\pm \sqrt{-\frac{\chi \left(ab_{1}\left(3m^{2}+5m+2\right)+2b_{2}\left(\alpha \left(m+4\right)-4\beta \right)\right)}{2ab_{2}{\left(m+1\right)}^{2}}}\text{,}$$

$$k=\frac{m}{2}\sqrt{-\frac{ab_{1}\left(3m^{2}+5m+2\right)+2b_{2}\left(\alpha \left(m+4\right)-4\beta \right)}{a^{2}{\left(m+1\right)}^{3}\left(3m+2\right)}}\text{,}$$

(56) $$\omega =\frac{a^{2}\gamma \left(3m+2\right){\left(m+1\right)}^{3}+4ab_{1}\left(3m^{2}+5m+2\right)\left(\alpha -\beta \right)+8b_{2}\left(\alpha -\beta \right)\left(\alpha \left(m+4\right)-4\beta \right)}{a^{2}{\left(m+1\right)}^{3}\left(3m+2\right)}\text{.}$$

The nonlinear wave profile is generated by plugging equation (56) with the assistance of equation (6) into equation (55), as extracted below

q(x,t)=

$${\left\{\begin{array}{c} \sqrt{-\frac{\chi \left(ab_{1}\left(3m^{2}+5m+2\right)+2b_{2}\left(\alpha \left(m+4\right)-4\beta \right)\right)}{2ab_{2}{\left(m+1\right)}^{2}}}\\ \times \;\frac{4c}{\left(\begin{array}{c} 4c^{2}\;exp\left[\frac{m}{2}\sqrt{-\frac{ab_{1}\left(3m^{2}+5m+2\right)+2b_{2}\left(\alpha \left(m+4\right)-4\beta \right)}{a^{2}{\left(m+1\right)}^{3}\left(3m+2\right)}}\;x\right]\\ +\chi \;exp\left[-\frac{m}{2}\sqrt{-\frac{ab_{1}\left(3m^{2}+5m+2\right)+2b_{2}\left(\alpha \left(m+4\right)-4\beta \right)}{a^{2}{\left(m+1\right)}^{3}\left(3m+2\right)}}\;x\right] \end{array}\right)} \end{array}\right\}}^{\frac{2}{m}}$$

(57) $$\times e^{i\left(\left\{\frac{a^{2}\gamma \left(3m+2\right){\left(m+1\right)}^{3}+4ab_{1}\left(3m^{2}+5m+2\right)\left(\alpha -\beta \right)+8b_{2}\left(\alpha -\beta \right)\left(\alpha \left(m+4\right)-4\beta \right)}{a^{2}{\left(m+1\right)}^{3}\left(3m+2\right)}\right\}t+\theta_{0}\right)}\text{.}$$

Solution (57) leads to the singular and bright solitons if we substitute $\chi =\pm 4c^{2}$, as formed below

$$q\left(x,t\right)={\left\{\pm \sqrt{\frac{ab_{1}\left(3m^{2}+5m+2\right)+2b_{2}\left(\alpha \left(m+4\right)-4\beta \right)}{2ab_{2}{\left(m+1\right)}^{2}}}\;\mathrm{csch}\left[\frac{m}{2}\sqrt{-\frac{ab_{1}\left(3m^{2}+5m+2\right)+2b_{2}\left(\alpha \left(m+4\right)-4\beta \right)}{a^{2}{\left(m+1\right)}^{3}\left(3m+2\right)}}\;x\right]\right\}}^{\frac{2}{m}}$$

$$\times e^{i\left(\left\{\frac{a^{2}\gamma \left(3m+2\right){\left(m+1\right)}^{3}+4ab_{1}\left(3m^{2}+5m+2\right)\left(\alpha -\beta \right)+8b_{2}\left(\alpha -\beta \right)\left(\alpha \left(m+4\right)-4\beta \right)}{a^{2}{\left(m+1\right)}^{3}\left(3m+2\right)}\right\}t+\theta_{0}\right)}\text{,}$$

and

$$q\left(x,t\right)={\left\{\pm \sqrt{-\frac{ab_{1}\left(3m^{2}+5m+2\right)+2b_{2}\left(\alpha \left(m+4\right)-4\beta \right)}{2ab_{2}{\left(m+1\right)}^{2}}}\mathrm{sech}\left[\frac{m}{2}\sqrt{-\frac{ab_{1}\left(3m^{2}+5m+2\right)+2b_{2}\left(\alpha \left(m+4\right)-4\beta \right)}{a^{2}{\left(m+1\right)}^{3}\left(3m+2\right)}}\;x\right]\right\}}^{\frac{2}{m}}$$

(58) $$\times e^{i\left(\left\{\frac{a^{2}\gamma \left(3m+2\right){\left(m+1\right)}^{3}+4ab_{1}\left(3m^{2}+5m+2\right)\left(\alpha -\beta \right)+8b_{2}\left(\alpha -\beta \right)\left(\alpha \left(m+4\right)-4\beta \right)}{a^{2}{\left(m+1\right)}^{3}\left(3m+2\right)}\right\}t+\theta_{0}\right)}\text{,}$$

where

$$ab_{1}\left(3m^{2}+5m+2\right)+2b_{2}\left(\alpha \left(m+4\right)-4\beta \right)<0\text{.}$$

**Fig. 5:**
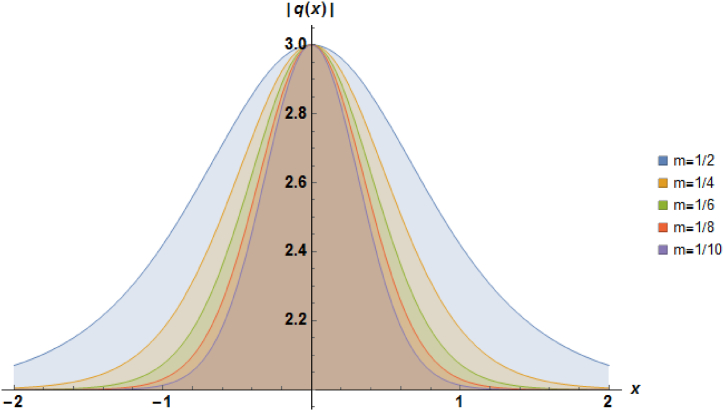
Characteristics of a stationary bright soliton profile.

Plots of the soliton described by equation (58) with β=−1, α=1, $b_{2}=-1$, a=1 and $b_{1}=-1$ are displayed in Fig. 5.

**Result–2**:

$$\lambda_{01}=0,\lambda_{0}=\pm \sqrt{\frac{-ab_{1}\left(3m^{2}+5m+2\right)-4\alpha b_{2}m}{4ab_{2}{\left(m+1\right)}^{2}}},\lambda_{10}=-2\eta \lambda_{0},k=m\sqrt{\frac{ab_{1}\left(3m^{2}+5m+2\right)+4\alpha b_{2}m}{2a^{2}{\left(m+1\right)}^{3}\left(3m+2\right)}}\text{,}$$

(59) $$\omega =\frac{16\left(a^{2}\gamma {\left(m+1\right)}^{4}+\alpha^{2}b_{2}m^{2}\right)}{16a^{2}{\left(m+1\right)}^{4}}+\frac{8\alpha b_{1}\left(m+1\right)m^{2}}{16a{\left(m+1\right)}^{4}}-\frac{b_{1}^{2}{\left(m+1\right)}^{2}\left(3m^{2}+8m+4\right)}{16b_{2}{\left(m+1\right)}^{4}},\beta =\alpha \left(1-\frac{m}{4}\right)\text{.}$$

Inserting (59) with the usage of (7), (55) allows us the optoelectronic wave field

$$q\left(x,t\right)={\left\{\pm \sqrt{\frac{-ab_{1}\left(3m^{2}+5m+2\right)-4\alpha b_{2}m}{4ab_{2}{\left(m+1\right)}^{2}}}\left(1-\frac{2\eta }{d\;\exp\left[m\sqrt{\frac{ab_{1}\left(3m^{2}+5m+2\right)+4\alpha b_{2}m}{2a^{2}{\left(m+1\right)}^{3}\left(3m+2\right)}}\;x\right]+\eta }\right)\right\}}^{\frac{2}{m}}$$

(60) $$\times e^{i\left(\left\{\frac{16\left(a^{2}\gamma {\left(m+1\right)}^{4}+\alpha^{2}b_{2}m^{2}\right)}{16a^{2}{\left(m+1\right)}^{4}}+\frac{8\alpha b_{1}\left(m+1\right)m^{2}}{16a{\left(m+1\right)}^{4}}-\frac{b_{1}^{2}{\left(m+1\right)}^{2}\left(3m^{2}+8m+4\right)}{16b_{2}{\left(m+1\right)}^{4}}\right\}t+\theta_{0}\right)}\text{.}$$

The dark and singular solitons can be derived from solution (60) by assuming η=±d, as recovered below

$$q\left(x,t\right)={\left\{\pm \sqrt{\frac{-ab_{1}\left(3m^{2}+5m+2\right)-4\alpha b_{2}m}{4ab_{2}{\left(m+1\right)}^{2}}}\tanh\left[m\sqrt{\frac{ab_{1}\left(3m^{2}+5m+2\right)+4\alpha b_{2}m}{8a^{2}{\left(m+1\right)}^{3}\left(3m+2\right)}}\;x\right]\right\}}^{\frac{2}{m}}$$

(61) $$\times e^{i\left(\left\{\frac{16\left(a^{2}\gamma {\left(m+1\right)}^{4}+\alpha^{2}b_{2}m^{2}\right)}{16a^{2}{\left(m+1\right)}^{4}}+\frac{8\alpha b_{1}\left(m+1\right)m^{2}}{16a{\left(m+1\right)}^{4}}-\frac{b_{1}^{2}{\left(m+1\right)}^{2}\left(3m^{2}+8m+4\right)}{16b_{2}{\left(m+1\right)}^{4}}\right\}t+\theta_{0}\right)}\text{,}$$

and

$$q\left(x,t\right)={\left\{\mp \sqrt{\frac{-ab_{1}\left(3m^{2}+5m+2\right)-4\alpha b_{2}m}{4ab_{2}{\left(m+1\right)}^{2}}}\coth\left[m\sqrt{\frac{ab_{1}\left(3m^{2}+5m+2\right)+4\alpha b_{2}m}{8a^{2}{\left(m+1\right)}^{3}\left(3m+2\right)}}\;x\right]\right\}}^{\frac{2}{m}}$$

$$\times e^{i\left(\left\{\frac{16\left(a^{2}\gamma {\left(m+1\right)}^{4}+\alpha^{2}b_{2}m^{2}\right)}{16a^{2}{\left(m+1\right)}^{4}}+\frac{8\alpha b_{1}\left(m+1\right)m^{2}}{16a{\left(m+1\right)}^{4}}-\frac{b_{1}^{2}{\left(m+1\right)}^{2}\left(3m^{2}+8m+4\right)}{16b_{2}{\left(m+1\right)}^{4}}\right\}t+\theta_{0}\right)}\text{,}$$

where

$$ab_{1}\left(3m^{2}+5m+2\right)+4\alpha b_{2}m>0\text{.}$$

**Fig. 6:**
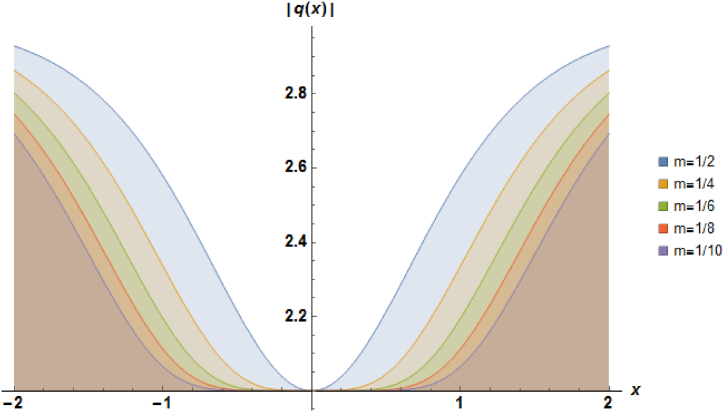
Characteristics of a stationary dark soliton profile.

Plots of the soliton described by equation (61) with a=1, $b_{1}=1$, α=1 and $b_{2}=1$ are shown in Fig. 6.

### 3.7 Cubic–quartic law

Eq. (8) stands as

$$iq_{t}+a{\left({\left|q\right|}^{n}q\right)}_{xx}+\left(b_{1}{\left|q\right|}^{2}+b_{2}{\left|q\right|}^{3}\right)q=\frac{1}{{\left|q\right|}^{2}q^{*}}\left\{\alpha {\left|q\right|}^{2}{\left({\left|q\right|}^{2}\right)}_{xx}-\beta {\left\{{\left({\left|q\right|}^{2}\right)}_{x}\right\}}^{2}\right\}+\gamma q\text{.}$$

In this case, Eq. (10) becomes

(62) $$ak^{2}\left(n+1\right)U^{n+1}U^{\prime \prime }+ak^{2}n\left(n+1\right)U^{n}{U^{\prime }}^{2}+b_{1}U^{4}+b_{2}U^{5}-2\alpha k^{2}UU^{\prime \prime }-2k^{2}\left(\alpha -2\beta \right){U^{\prime }}^{2}+\left(\gamma -\omega \right)U^{2}=0\text{.}$$

For integrability, we set n=2. So (62) changes to

(63) $$3ak^{2}U^{3}U^{\prime \prime 2}U^{2}U^{\prime 2}+b_{2}U^{5}+b_{1}U^{4}-2\alpha k^{2}UU^{\prime \prime 2}\left(\alpha -2\beta \right)U^{\prime 2}+\left(\gamma -\omega \right)U^{2}=0\text{.}$$

Balancing $U^{3}U^{\prime \prime }$ with $U^{5}$ in Eq. (63) gives N=2. Thus, we arrive at

(64) $$U=\lambda_{0}+\lambda_{01}R+\lambda_{10}Q+\lambda_{11}RQ+\lambda_{02}R^{2}+\lambda_{20}Q^{2}\text{.}$$

Inserting (64) along with (4), (5), (63) yields a polynomial of Q,R and $R^{\prime }$. We can solve the system of equations that arises when we set all terms with the same powers to zero. The solutions reveal the following outcomes:

Result**–1**:

(65) $$\lambda_{0}=\lambda_{01}=\lambda_{11}=\lambda_{20}=0,\lambda_{02}=-\frac{7b_{1}\chi }{6b_{2}},k=\pm \frac{1}{6}\sqrt{-\frac{b_{1}}{a}},\omega =\gamma -\frac{\alpha b_{1}}{9a},\beta =\frac{5\alpha }{4}\text{.}$$

The soliton wave profile emerges when we insert equation (65) with (6) into equation (64), as defined below

$$q\left(x,t\right)=-\frac{7b_{1}\chi }{6b_{2}}{\left(\frac{4c}{4c^{2}\;\exp\left[\pm \frac{1}{6}\sqrt{-\frac{b_{1}}{a}}\;x\right]+\chi \;\exp\left[\mp \frac{1}{6}\sqrt{-\frac{b_{1}}{a}}\;x\right]}\right)}^{2}$$

(66) $$\times e^{i\left(\left\{\gamma -\frac{\alpha b_{1}}{9a}\right\}t+\theta_{0}\right)}\text{.}$$

The evolution of the quiescent singular and bright solitons can be derived from solution (66) by using $\chi =\pm 4c^{2}$, as presented below

$$q\left(x,t\right)=\frac{7b_{1}}{6b_{2}}\csc\;h^{2}\left[\frac{1}{6}\sqrt{-\frac{b_{1}}{a}}\;x\right]e^{i\left(\left\{\gamma -\frac{\alpha b_{1}}{9a}\right\}t+\theta_{0}\right)}\text{,}$$

and

$$q\left(x,t\right)=-\frac{7b_{1}}{6b_{2}}\sec\;h^{2}\left[\frac{1}{6}\sqrt{-\frac{b_{1}}{a}}\;x\right]e^{i\left(\left\{\gamma -\frac{\alpha b_{1}}{9a}\right\}t+\theta_{0}\right)}\text{,}$$

respectively. The range of applicability for these solitons is

$$ab_{1}<0\text{.}$$

**Result–2**:

(67) $$\lambda_{0}=\lambda_{01}=\lambda_{11}=\lambda_{02}=0,\lambda_{10}=-\frac{14b_{1}\eta }{3b_{2}},\lambda_{20}=-\eta \lambda_{10},k=\pm \frac{1}{3}\sqrt{-\frac{b_{1}}{a}},\omega =\gamma -\frac{\alpha b_{1}}{9a},\beta =\frac{5\alpha }{4}\text{.}$$

Inserting (67) along with (6), (64) provides us the nonlinear waveform

(68) $$q\left(x,t\right)=-\frac{14b_{1}d\eta \;\exp\left[\pm \frac{1}{3}\sqrt{-\frac{b_{1}}{a}}\;x\right]}{3b_{2}{\left(d\;\exp\left[\pm \frac{1}{3}\sqrt{-\frac{b_{1}}{a}}\;x\right]+\eta \right)}^{2}}e^{i\left(\left\{\gamma -\frac{\alpha b_{1}}{9a}\right\}t+\theta_{0}\right)}\text{.}$$

The quiescent singular and bright solitons arise from solution (68) when we set η=±d, as shown below:

$$q\left(x,t\right)=\frac{7b_{1}}{6b_{2}}\csc\;h^{2}\left[\frac{1}{6}\sqrt{-\frac{b_{1}}{a}}\;x\right]e^{i\left(\left\{\gamma -\frac{\alpha b_{1}}{9a}\right\}t+\theta_{0}\right)}\text{,}$$

and

$$q\left(x,t\right)=-\frac{7b_{1}}{6b_{2}}\sec\;h^{2}\left[\frac{1}{6}\sqrt{-\frac{b_{1}}{a}}\;x\right]e^{i\left(\left\{\gamma -\frac{\alpha b_{1}}{9a}\right\}t+\theta_{0}\right)}\text{,}$$

respectively. These solitons are valid for

$$ab_{1}<0\text{.}$$

### 3.8 Generalized cubic–quartic law

Equation (8) takes the form of

$$iq_{t}+a{\left({\left|q\right|}^{n}q\right)}_{xx}+\left(b_{1}{\left|q\right|}^{2m}+b_{2}{\left|q\right|}^{3m}\right)q=\frac{1}{{\left|q\right|}^{2}q^{*}}\left\{\alpha {\left|q\right|}^{2}{\left({\left|q\right|}^{2}\right)}_{xx}-\beta {\left\{{\left({\left|q\right|}^{2}\right)}_{x}\right\}}^{2}\right\}+\gamma q\text{.}$$

In this case, Eq. (10) becomes

$$ak^{2}\left(n+1\right)U^{n+1}U^{\prime \prime }+ak^{2}n\left(n+1\right)U^{n}{U^{\prime }}^{2}+b_{1}U^{2m+2}+b_{2}U^{3m+2}-2\alpha k^{2}UU^{\prime \prime }-2k^{2}\left(\alpha -2\beta \right){U^{\prime }}^{2}+$$

(69) $$\left(\gamma -\omega \right)U^{2}=0\text{.}$$

We can choose n=2m. So Eq. (69) changes to

$$ak^{2}{\left(2m+1\right)}^{2m+1}U^{\prime \prime 2}m\left(2m+1\right)U^{2m}U^{\prime 2}+b_{1}U^{2m+2}+b_{2}U^{3m+2}-2\alpha k^{2}UU^{\prime \prime 2}\left(\alpha -2\beta \right)U^{\prime 2}+$$

(70) $$\left(\gamma -\omega \right)U^{2}=0\text{.}$$

Now, we assume

$$U=V^{\frac{2}{m}}\text{.}$$

Thus, Eq. (70) shapes up as

$$2k^{2}V^{\prime 2}\left(a\left(6m^{2}+7m+2\right)V^{4}+8\beta +2\alpha \left(m-4\right)\right)+2k^{2}mVV^{\prime \prime }\left(a\left(2m+1\right)V^{4}-2\alpha \right)+$$

(71) $$m^{2}V^{2}\left(b_{2}V^{6}+b_{1}V^{4}+\gamma -\omega \right)=0\text{.}$$

If we balance $V^{8}$ with $V^{5}V^{\prime \prime }$ in Eq. (71), we obtain N=1, which leads to the outcome:

(72) $$V=\lambda_{0}+\lambda_{01}R+\lambda_{10}Q\text{.}$$

Substituting (72) along with (4), (5), (71) leads to a polynomial of Q,R and $R^{\prime }$. Collecting terms with the same powers and equating them to zero leads to a set of equations that can be addressed to derive the findings:

**Result–1**:

$$\lambda_{0}=\lambda_{10}=0,\lambda_{01}=\pm \sqrt{-\frac{b_{1}\left(5m+2\right)\chi }{b_{2}\left(4m+2\right)}},k=\frac{1}{2}m\sqrt{-\frac{b_{1}}{a{\left(2m+1\right)}^{2}}}\text{,}$$

(73) $$\omega =\frac{a\gamma {\left(2m+1\right)}^{2}-\alpha b_{1}m}{a{\left(2m+1\right)}^{2}},\beta =\frac{1}{4}\alpha \left(m+4\right)\text{.}$$

Plugging (73) with the aid of (6), (72) allows us the nonlinear wave profile

$$q\left(x,t\right)={\left\{\pm \frac{4c\sqrt{-\frac{b_{1}\left(5m+2\right)\chi }{b_{2}\left(4m+2\right)}}}{4c^{2}\;\exp\left[\frac{m}{2}\sqrt{-\frac{b_{1}}{a{\left(2m+1\right)}^{2}}}\;x\right]+\chi \;\exp\left[-\frac{m}{2}\sqrt{-\frac{b_{1}}{a{\left(2m+1\right)}^{2}}}\;x\right]}\right\}}^{\frac{2}{m}}$$

(74) $$\times e^{i\left(\left\{\frac{a\gamma {\left(2m+1\right)}^{2}-\alpha b_{1}m}{a{\left(2m+1\right)}^{2}}\right\}t+\theta_{0}\right)}\text{.}$$

Choosing $\chi =\pm 4c^{2}$ in solution (74) provides us the quiescent singular and bright solitons

$$q\left(x,t\right)={\left\{\pm \sqrt{\frac{b_{1}\left(5m+2\right)}{b_{2}\left(4m+2\right)}}\mathrm{csch}\left[\frac{m}{2}\sqrt{-\frac{b_{1}}{a{\left(2m+1\right)}^{2}}}\;x\right]\right\}}^{\frac{2}{m}}$$

$$\times e^{i\left(\left\{\frac{a\gamma {\left(2m+1\right)}^{2}-\alpha b_{1}m}{a{\left(2m+1\right)}^{2}}\right\}t+\theta_{0}\right)}\text{,}$$

and

$$q\left(x,t\right)={\left\{\pm \sqrt{-\frac{b_{1}\left(5m+2\right)}{b_{2}\left(4m+2\right)}}\mathrm{sech}\left[\frac{m}{2}\sqrt{-\frac{b_{1}}{a{\left(2m+1\right)}^{2}}}\;x\right]\right\}}^{\frac{2}{m}}$$

(75) $$\times e^{i\left(\left\{\frac{a\gamma {\left(2m+1\right)}^{2}-\alpha b_{1}m}{a{\left(2m+1\right)}^{2}}\right\}t+\theta_{0}\right)}\text{,}$$

respectively. These solitons are valid for

$$ab_{1}<0\text{.}$$

**Fig. 7:**
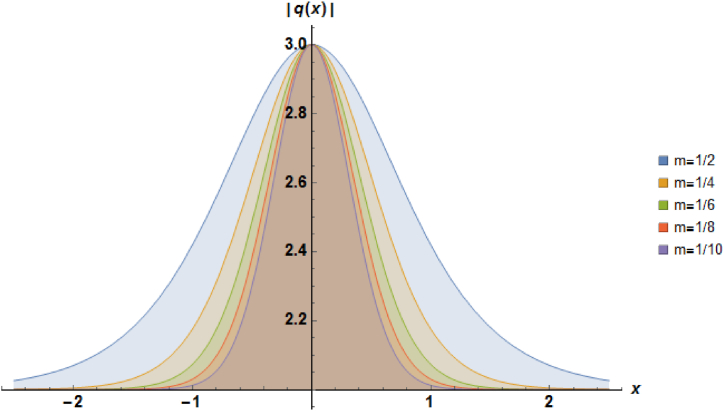
Characteristics of a stationary bright soliton profile.

Plots of the soliton described by equation (75) with a=1, $b_{1}=-1$ and $b_{2}=1$ are illustrated in Fig. 7.

**Result–2**:

$$\lambda_{01}=\lambda_{0}=\pm \sqrt{-2}\sqrt[{4}]{\frac{\alpha m}{a\left(6m^{2}+7m+2\right)}},\lambda_{10}=-2\eta \lambda_{0},k=\sqrt[{4}]{\frac{4\alpha b_{2}^{2}m^{5}}{a^{3}\left(6m^{2}+7m+2\right){\left(10m^{2}+9m+2\right)}^{2}}}\text{,}$$

$$\omega =\frac{8b_{2}\sqrt{\alpha^{3}m^{3}}}{a\left(10m^{2}+9m+2\right)\sqrt{a\left(6m^{2}+7m+2\right)}}+\gamma ,\beta =\alpha \left(1-\frac{m}{4}\right)\text{,}$$

(76) $$b_{1}=\frac{8b_{2}\left(2m+1\right)\sqrt{\alpha m}}{\left(5m+2\right)\sqrt{a\left(6m^{2}+7m+2\right)}}\text{.}$$

Putting (76) with the help of (7), (72) causes to the optoelectronic wave field

$$q\left(x,t\right)={\left\{\pm \sqrt{-2}\sqrt[{4}]{\frac{\alpha m}{a\left(6m^{2}+7m+2\right)}}\left(1-\frac{2\eta }{d\;\exp\left[\sqrt[{4}]{\frac{4\alpha b_{2}^{2}m^{5}}{a^{3}\left(6m^{2}+7m+2\right){\left(10m^{2}+9m+2\right)}^{2}}}\;x\right]+\eta }\right)\right\}}^{\frac{2}{m}}$$

(77) $$\times e^{i\left(\left\{\frac{8b_{2}\sqrt{\alpha^{3}m^{3}}}{a\left(10m^{2}+9m+2\right)\sqrt{a\left(6m^{2}+7m+2\right)}}+\gamma \right\}t+\theta_{0}\right)}\text{.}$$

The solution (77) yields quiescent dark and singular solitons if we assume η to be equal to ±d, as presented below:

$$q\left(x,t\right)={\left\{\pm \sqrt{-2}\sqrt[{4}]{\frac{\alpha m}{a\left(6m^{2}+7m+2\right)}}\tanh\left[\sqrt[{4}]{\frac{\alpha b_{2}^{2}m^{5}}{4a^{3}\left(6m^{2}+7m+2\right){\left(10m^{2}+9m+2\right)}^{2}}}\;x\right]\right\}}^{\frac{2}{m}}$$

(78) $$\times e^{i\left(\left\{\frac{8b_{2}\sqrt{\alpha^{3}m^{3}}}{a\left(10m^{2}+9m+2\right)\sqrt{a\left(6m^{2}+7m+2\right)}}+\gamma \right\}t+\theta_{0}\right)}\text{,}$$

and

$$q\left(x,t\right)={\left\{\mp \sqrt{-2}\sqrt[{4}]{\frac{\alpha m}{a\left(6m^{2}+7m+2\right)}}\coth\left[\sqrt[{4}]{\frac{\alpha b_{2}^{2}m^{5}}{4a^{3}\left(6m^{2}+7m+2\right){\left(10m^{2}+9m+2\right)}^{2}}}\;x\right]\right\}}^{\frac{2}{m}}$$

$$\times e^{i\left(\left\{\frac{8b_{2}\sqrt{\alpha^{3}m^{3}}}{a\left(10m^{2}+9m+2\right)\sqrt{a\left(6m^{2}+7m+2\right)}}+\gamma \right\}t+\theta_{0}\right)}\text{,}$$

respectively. These solitons are valid for

aα>0.

**Fig. 8:**
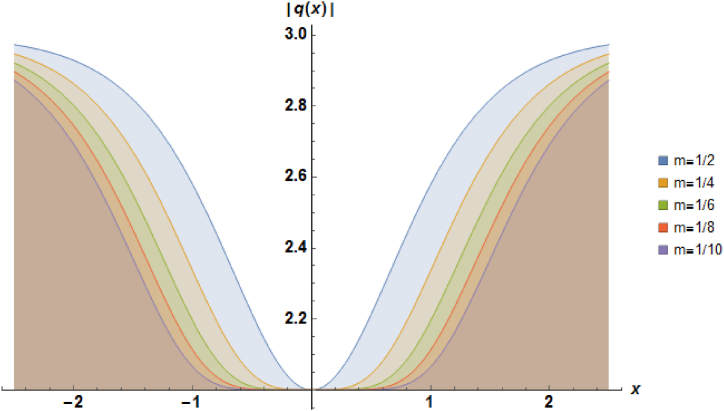
Characteristics of a stationary dark soliton profile.

Plots of the soliton described by equation (78) with a=1, α=1 and $b_{2}=1$ are demonstrated in Fig. 8.

### 3.9 Polynomial law

Simplification of (8) yields

$$iq_{t}+a{\left({\left|q\right|}^{n}q\right)}_{xx}+\left(b_{1}{\left|q\right|}^{2}+b_{2}{\left|q\right|}^{4}+b_{3}{\left|q\right|}^{6}\right)q=\frac{1}{{\left|q\right|}^{2}q^{*}}\left\{\alpha {\left|q\right|}^{2}{\left({\left|q\right|}^{2}\right)}_{xx}-\beta {\left\{{\left({\left|q\right|}^{2}\right)}_{x}\right\}}^{2}\right\}+\gamma q\text{.}$$

Thus, Eq. (10) simplifies to

(79) $$ak^{2}\left(n+1\right)U^{n+1}U^{\prime \prime }+ak^{2}n\left(n+1\right)U^{n}{U^{\prime }}^{2}+b_{1}U^{4}+b_{2}U^{6}+b_{3}U^{8}-2\alpha k^{2}UU^{\prime \prime }-2k^{2}\left(\alpha -2\beta \right){U^{\prime }}^{2}+\left(\gamma -\omega \right)U^{2}=0\text{.}$$

For integrability, we set n=4. So Eq. (79) changes to

(80) $$5ak^{2}U^{5}U^{\prime \prime 2}U^{4}U^{\prime 2}+b_{3}U^{8}+b_{2}U^{6}+b_{1}U^{4}-2\alpha k^{2}UU^{\prime \prime 2}\left(\alpha -2\beta \right)U^{\prime 2}+\left(\gamma -\omega \right)U^{2}=0\text{.}$$

Balancing $U^{5}U^{\prime \prime }$ with $U^{8}$ in Eq. (80) gives N=1. Therefore, we arrive at

(81) $$U=\lambda_{0}+\lambda_{01}R+\lambda_{10}Q\text{.}$$

Plugging (81) along with (4), (5), (80) enables us a polynomial of Q,R and $R^{\prime }$. We can obtain a system of equations by gathering all terms with the same powers and setting them to zero. Solving this system allows us to secure the following outcomes:

**Result–1**:

(82) $$\lambda_{0}=\lambda_{10}=0,\lambda_{01}=\pm \sqrt{-\frac{6b_{2}\chi }{5b_{3}}},k=\frac{1}{5}\sqrt{-\frac{b_{2}}{a}},\omega =\frac{4b_{2}\left(\alpha -\beta \right)}{25a}+\gamma ,b_{1}=\frac{b_{3}\left(2\beta -3\alpha \right)}{15a}\text{.}$$

The soliton wave profile can be obtained by plugging (82) with (6), (81), as seen below:

(83) $$q\left(x,t\right)=\pm \frac{4c\sqrt{-\frac{6b_{2}\chi }{5b_{3}}}}{4c^{2}\;\exp\left[\frac{1}{5}\sqrt{-\frac{b_{2}}{a}}\;x\right]+\chi \;\exp\left[-\frac{1}{5}x\sqrt{-\frac{b_{2}}{a}}\right]}\;e^{i\left(\left\{\frac{4b_{2}\left(\alpha -\beta \right)}{25a}+\gamma \right\}t+\theta_{0}\right)}\text{.}$$

Setting $\chi =\pm 4c^{2}$ in solution (83) leads to the quiescent singular and bright solitons

$$q\left(x,t\right)=\pm \sqrt{\frac{6b_{2}}{5b_{3}}}\mathrm{csch}\left[\frac{1}{5}\sqrt{-\frac{b_{2}}{a}}x\right]e^{i\left(\left\{\frac{4b_{2}\left(\alpha -\beta \right)}{25a}+\gamma \right\}t+\theta_{0}\right)}\text{,}$$

and

$$q\left(x,t\right)=\pm \sqrt{-\frac{6b_{2}}{5b_{3}}}\mathrm{sech}\left[\frac{1}{5}\sqrt{-\frac{b_{2}}{a}}\;x\right]e^{i\left(\left\{\frac{4b_{2}\left(\alpha -\beta \right)}{25a}+\gamma \right\}t+\theta_{0}\right)}\text{,}$$

respectively. The range of validity for these solitons is

$$ab_{2}<0\text{.}$$

**Result–2**:

(84) $$\lambda_{01}=\lambda_{0}=\pm \sqrt{-\frac{3b_{2}}{5b_{3}}},\lambda_{10}=-2\eta \lambda_{0},k=\frac{1}{5}\sqrt{\frac{2b_{2}}{a}},\omega =\frac{2\alpha b_{2}}{25a}+\gamma ,b_{1}=\frac{6b_{2}^{2}}{25b_{3}}-\frac{2\alpha b_{3}}{15a},\beta =\frac{\alpha }{2}\text{.}$$

Putting (84) with the aid of (7), (81) provides us the nonlinear waveform

(85) $$q\left(x,t\right)=\pm \sqrt{-\frac{3b_{2}}{5b_{3}}}\left(1-\frac{2\eta }{d\;\exp\left[\frac{1}{5}\sqrt{\frac{2b_{2}}{a}}\;x\right]+\eta }\right)e^{i\left(\left\{\frac{2\alpha b_{2}}{25a}+\gamma \right\}t+\theta_{0}\right)}\text{.}$$

The quiescent singular and dark solitons can be obtained from solution (85) by setting η equal to ±d, as shown below:

$$q\left(x,t\right)=\mp \sqrt{-\frac{3b_{2}}{5b_{3}}}\coth\left[\frac{1}{10}\sqrt{\frac{2b_{2}}{a}}\;x\right]e^{i\left(\left\{\frac{2\alpha b_{2}}{25a}+\gamma \right\}t+\theta_{0}\right)}\text{,}$$

and

$$q\left(x,t\right)=\pm \sqrt{-\frac{3b_{2}}{5b_{3}}}\tanh\left[\frac{1}{10}\sqrt{\frac{2b_{2}}{a}}\;x\right]e^{i\left(\left\{\frac{2\alpha b_{2}}{25a}+\gamma \right\}t+\theta_{0}\right)}\text{,}$$

respectively. These solitons are valid for

$$ab_{2}>0\text{.}$$

### 3.10 Triple–power law

Eq. (8) falls out as

$$iq_{t}+a{\left({\left|q\right|}^{n}q\right)}_{xx}+\left(b_{1}{\left|q\right|}^{2m}+b_{2}{\left|q\right|}^{4m}+b_{3}{\left|q\right|}^{6m}\right)q=\frac{1}{{\left|q\right|}^{2}q^{*}}\left\{\alpha {\left|q\right|}^{2}{\left({\left|q\right|}^{2}\right)}_{xx}-\beta {\left\{{\left({\left|q\right|}^{2}\right)}_{x}\right\}}^{2}\right\}+\gamma q\text{.}$$

After the substitution, Eq. (10) can be expressed as:

$$ak^{2}\left(n+1\right)U^{n+1}U^{\prime \prime }+ak^{2}n\left(n+1\right)U^{n}{U^{\prime }}^{2}+b_{1}U^{2m+2}+b_{2}U^{4m+2}++b_{3}U^{6m+2}-$$

$$2\alpha k^{2}UU^{\prime \prime }-2k^{2}\left(\alpha -2\beta \right){U^{\prime }}^{2}+$$

(86) $$\left(\gamma -\omega \right)U^{2}=0\text{.}$$

We can choose n=4m. So Eq. (86) changes to

$$ak^{2}\left(4m+1\right)U^{4m+1}U^{\prime \prime 2}m\left(4m+1\right)U^{4m}U^{\prime 2}+b_{1}U^{2m+2}+b_{2}U^{4m+2}+b_{3}U^{6m+2}-$$

(87) $$2\alpha k^{2}UU^{\prime \prime 2}\left(\alpha -2\beta \right)U^{\prime 2}+\left(\gamma -\omega \right)U^{2}=0\text{.}$$

Now, we assume

$$U=V^{\frac{1}{m}}\text{.}$$

Thus, Eq. (87) reduces to

$$k^{2}V^{\prime 2}\left(a\left(12m^{2}+7m+1\right)V^{4}+4\beta +2\alpha \left(m-2\right)\right)+k^{2}mVV^{\prime \prime }\left(a\left(4m+1\right)V^{4}-2\alpha \right)+$$

(88) $$m^{2}V^{2}\left(b_{3}V^{6}+b_{2}V^{4}+b_{1}V^{2}+\gamma -\omega \right)=0\text{.}$$

Balancing $V^{8}$ with $V^{5}V^{\prime \prime }$ in Eq. (88) enables us N=1. In this case, we arrive at

(89) $$V=\lambda_{0}+\lambda_{01}R+\lambda_{10}Q\text{.}$$

Inserting (89) along with (4), (5), (88) provides us a polynomial of Q,R and $R^{\prime }$. By grouping all the terms with the same powers and setting them equal to zero, we can derive a system of equations that allows us to secure the results:

**Result–1**:

$$\lambda_{0}=\lambda_{10}=0,\lambda_{01}=\pm \sqrt{-\frac{b_{2}\left(5m+1\right)\chi }{b_{3}\left(4m+1\right)}},k=m\sqrt{-\frac{b_{2}}{a{\left(4m+1\right)}^{2}}},\omega =\frac{4b_{2}\left(\alpha -\beta \right)}{a{\left(4m+1\right)}^{2}}+\gamma \text{,}$$

(90) $$b_{1}=-\frac{2b_{3}\left(\alpha \left(m+2\right)-2\beta \right)}{a\left(20m^{2}+9m+1\right)}\text{.}$$

Substituting (90), using the relation given in (6), into Eq. (89) results in the following nonlinear wave profile:

$$q\left(x,t\right)={\left\{\pm \frac{4c\sqrt{-\frac{b_{2}\left(5m+1\right)\chi }{b_{3}\left(4m+1\right)}}}{4c^{2}\;\exp\left[m\sqrt{-\frac{b_{2}}{a{\left(4m+1\right)}^{2}}}\;x\right]+\chi \;\exp\left[-m\sqrt{-\frac{b_{2}}{a{\left(4m+1\right)}^{2}}}\;x\right]}\right\}}^{\frac{1}{m}}$$

(91) $$\times e^{i\left(\left\{\frac{4b_{2}\left(\alpha -\beta \right)}{a{\left(4m+1\right)}^{2}}+\gamma \right\}t+\theta_{0}\right)}\text{.}$$

Choosing $\chi =\pm 4c^{2}$ in solution (91) gives way to the quiescent singular and bright solitons

$$q\left(x,t\right)={\left\{\pm \sqrt{\frac{b_{1}\left(5m+2\right)}{b_{2}\left(4m+2\right)}}\mathrm{csch}\left[\frac{m}{2}\sqrt{-\frac{b_{1}}{a{\left(2m+1\right)}^{2}}}\;x\right]\right\}}^{\frac{1}{m}}$$

$$\times e^{i\left(\left\{\frac{4b_{2}\left(\alpha -\beta \right)}{a{\left(4m+1\right)}^{2}}+\gamma \right\}t+\theta_{0}\right)}\text{,}$$

and

$$q\left(x,t\right)={\left\{\pm \sqrt{-\frac{b_{1}\left(5m+2\right)}{b_{2}\left(4m+2\right)}}\mathrm{sech}\left[\frac{m}{2}\sqrt{-\frac{b_{1}}{a{\left(2m+1\right)}^{2}}}\;x\right]\right\}}^{\frac{1}{m}}$$

(92) $$\times e^{i\left(\left\{\frac{4b_{2}\left(\alpha -\beta \right)}{a{\left(4m+1\right)}^{2}}+\gamma \right\}t+\theta_{0}\right)}\text{,}$$

respectively. These solitons are valid for

$$ab_{1}<0\text{.}$$

**Fig. 9:**
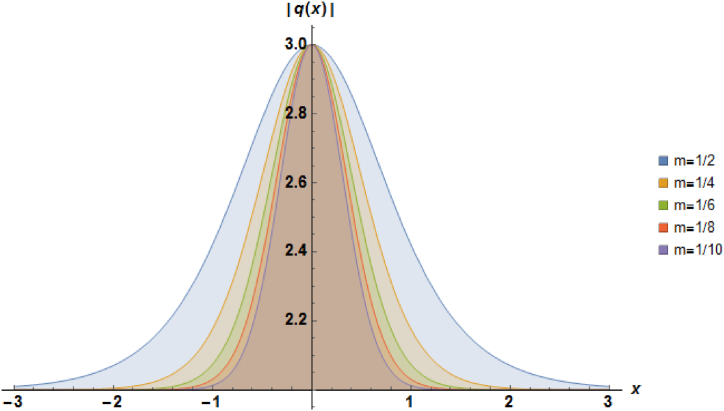
Characteristics of a stationary bright soliton profile.

Plots of the soliton described by equation (92) with a=1, $b_{1}=-1$ and $b_{2}=1$ are depicted in Fig. 9.

**Result–2**:

$$\lambda_{01}=\lambda_{0}=\pm \sqrt{-\frac{b_{2}\left(5m+1\right)}{2b_{3}\left(4m+1\right)}},\lambda_{10}=-2\eta \lambda_{0},k=m\sqrt{\frac{2b_{2}}{a{\left(4m+1\right)}^{2}}},\omega =\frac{a\gamma {\left(4m+1\right)}^{2}+2\alpha b_{2}m}{a{\left(4m+1\right)}^{2}}\text{,}$$

(93) $$b_{1}=\frac{b_{2}^{2}\left(3m+1\right)\left(5m+1\right)}{4b_{3}{\left(4m+1\right)}^{2}}-\frac{4\alpha b_{3}m}{20am^{2}+9am+a},\beta =\alpha \left(1-\frac{m}{2}\right)\text{.}$$

Putting (93) together with (7), (89) paves way to the soliton wave profile

$$q\left(x,t\right)={\left\{\pm \sqrt{-\frac{b_{2}\left(5m+1\right)}{2b_{3}\left(4m+1\right)}}\left(1-\frac{2\eta }{d\;\exp\left[m\sqrt{\frac{2b_{2}}{a{\left(4m+1\right)}^{2}}}\;x\right]+\eta }\right)\right\}}^{\frac{1}{m}}$$

(94) $$\times e^{i\left(\left\{\frac{a\gamma {\left(4m+1\right)}^{2}+2\alpha b_{2}m}{a{\left(4m+1\right)}^{2}}\right\}t+\theta_{0}\right)}\text{.}$$

When η=±d, the solutions (94) yield quiescent dark and singular solitons

$$q\left(x,t\right)={\left\{\pm \sqrt{-\frac{b_{2}\left(5m+1\right)}{2b_{3}\left(4m+1\right)}}\tanh\left[\frac{m}{2}\sqrt{\frac{2b_{2}}{a{\left(4m+1\right)}^{2}}}\;x\right]\right\}}^{\frac{1}{m}}$$

(95) $$\times e^{i\left(\left\{\frac{a\gamma {\left(4m+1\right)}^{2}+2\alpha b_{2}m}{a{\left(4m+1\right)}^{2}}\right\}t+\theta_{0}\right)}\text{,}$$

and

$$q\left(x,t\right)={\left\{\mp \sqrt{-\frac{b_{2}\left(5m+1\right)}{2b_{3}\left(4m+1\right)}}\coth\left[\frac{m}{2}\sqrt{\frac{2b_{2}}{a{\left(4m+1\right)}^{2}}}\;x\right]\right\}}^{\frac{1}{m}}$$

$$\times e^{i\left(\left\{\frac{a\gamma {\left(4m+1\right)}^{2}+2\alpha b_{2}m}{a{\left(4m+1\right)}^{2}}\right\}t+\theta_{0}\right)}\text{,}$$

respectively. These solitons are valid for

$$ab_{2}>0\text{.}$$

**Fig. 10:**
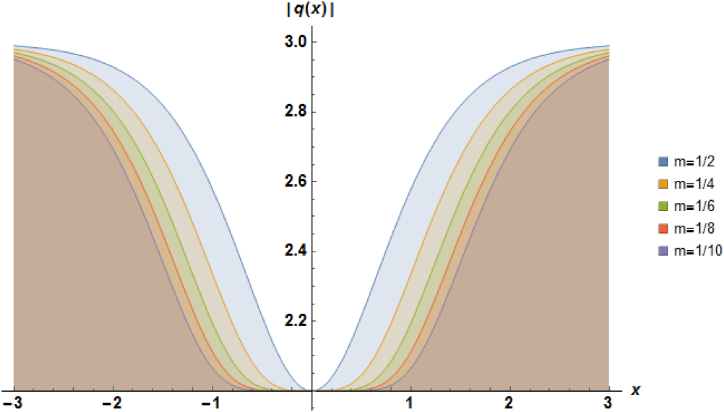
Characteristics of a stationary dark soliton profile.

Plots of the soliton described by equation (95) with a=1, $b_{2}=1$, and $b_{3}=-1$ are presented in Fig. 10.

### 3.11 Anti–cubic law

Eq. (8) reads as

$$iq_{t}+a{\left({\left|q\right|}^{n}q\right)}_{xx}+\left(\frac{b_{1}}{{\left|q\right|}^{4}}+b_{2}{\left|q\right|}^{2}+b_{3}{\left|q\right|}^{4}\right)q=\frac{1}{{\left|q\right|}^{2}q^{*}}\left\{\alpha {\left|q\right|}^{2}{\left({\left|q\right|}^{2}\right)}_{xx}-\beta {\left\{{\left({\left|q\right|}^{2}\right)}_{x}\right\}}^{2}\right\}+\gamma q\text{.}$$

Therefore, Eq. (10) can be transformed into the following form:

$$U^{2}(ak^{2}\left(n+1\right)U^{n+1}U^{\prime \prime 2}n\left(n+1\right)U^{n}U^{\prime 2}+b_{3}U^{6}+b_{2}U^{4}-2\alpha k^{2}UU^{\prime \prime }-$$

(96) $$2k^{2}\left(\alpha -2\beta \right)U^{\prime 2}+\left(\gamma -\omega \right)U^{2})+b_{1}=0\text{.}$$

For integrability, we set n=4. So Eq. (96) changes to

(97) $$U^{2}\left(3ak^{2}U^{3}U^{\prime \prime 2}U^{2}U^{\prime 2}+b_{3}U^{6}+b_{2}U^{4}-2\alpha k^{2}UU^{\prime \prime 2}\left(\alpha -2\beta \right)U^{\prime 2}+\left(\gamma -\omega \right)U^{2}\right)+b_{1}=0\text{.}$$

Balancing $U^{5}U^{\prime \prime }$ with $U^{8}$ in Eq. (97) gives N=1. Thus, we arrive at

(98) $$U=\lambda_{0}+\lambda_{01}R+\lambda_{10}Q\text{.}$$

Inserting (98) along with (4), (5), (97) leads to a polynomial in Q, R and $R^{\prime }$. We gather all terms that have the same exponents and set their sum equal to zero. Then, the resulting system of equations is solved by Mathematica to derive that $b_{1}=0$. This shows that the CGLE with anti–cubic nonlinearity collapses to parabolic law.

### 3.12 Generalized anti–cubic law

Simplifying Eq. (8) yields

$$iq_{t}+a{\left({\left|q\right|}^{n}q\right)}_{xx}+\left(\frac{b_{1}}{{\left|q\right|}^{2m+2}}+b_{2}{\left|q\right|}^{2m}+b_{3}{\left|q\right|}^{2m+2}\right)q=\frac{1}{{\left|q\right|}^{2}q^{*}}\left\{\alpha {\left|q\right|}^{2}{\left({\left|q\right|}^{2}\right)}_{xx}-\beta {\left\{{\left({\left|q\right|}^{2}\right)}_{x}\right\}}^{2}\right\}+\gamma q\text{.}$$

Thus, we can rewrite Eq. (10) as follows:

$$U^{2m}(ak^{2}\left(n+1\right)U^{n+1}U^{\prime \prime 2}n\left(n+1\right)U^{n}U^{\prime 2}+b_{2}U^{2m+2}+b_{3}U^{2m+4}-2\alpha k^{2}UU^{\prime \prime }-$$

(99) $$2k^{2}\left(\alpha -2\beta \right)U^{\prime 2}+\left(\gamma -\omega \right)U^{2})+b_{1}=0\text{.}$$

We can choose n=2m, but the results obtained in this case are the same for anti-cubic and parabolic law cases. So, we can choose n=m+1, then Eq. (99) changes to

$$U^{2m}(ak^{2}\left(m+2\right)U^{m+2}U^{\prime \prime 2}\left(m+1\right)\left(m+2\right)U^{m+1}U^{\prime 2}+b_{2}U^{2m+2}+b_{3}U^{2m+4}-2\alpha k^{2}UU^{\prime \prime }-$$

(100) $$2k^{2}\left(\alpha -2\beta \right)U^{\prime 2}+\left(\gamma -\omega \right)U^{2})+b_{1}=0\text{.}$$

Now, we assume

$$U=V^{\frac{2}{m+1}}\text{.}$$

Thus, Eq. (100) simplifies to

(101) $$V^{2}\left(2ak^{2}\left(m^{2}+3m+2\right)V^{3}V^{\prime \prime 2}\left(2ak^{2}\left(m^{2}+5m+6\right)V^{\prime 2}+{\left(m+1\right)}^{2}\left(\gamma -\omega \right)\right)+b_{2}{\left(m+1\right)}^{2}V^{\frac{4m}{m+1}+2}+b_{3}{\left(m+1\right)}^{2}V^{6}-4\alpha k^{2}\left(m+1\right)VV^{\prime \prime 2}\left(4\beta +\alpha \left(m-3\right)\right)V^{\prime 2}\right)+b_{1}{\left(m+1\right)}^{2}=0\text{.}$$

For integrability, we set $b_{2}=0$. Now Eq. (101) turns out to be

(102) $$V^{2}\left(2ak^{2}\left(m^{2}+3m+2\right)V^{3}V^{\prime \prime 2}\left(2ak^{2}\left(m^{2}+5m+6\right)V^{\prime 2}+{\left(m+1\right)}^{2}\left(\gamma -\omega \right)\right)+b_{3}{\left(m+1\right)}^{2}V^{6}-4\alpha k^{2}\left(m+1\right)VV^{\prime \prime 2}\left(4\beta +\alpha \left(m-3\right)\right)V^{\prime 2}\right)+b_{1}{\left(m+1\right)}^{2}=0\text{.}$$

By balancing the terms $V^{8}$ and $V^{5}V^{\prime \prime }$ in Eq. (102), we find that N must be equal to 1. As a result, we arrive at

(103) $$V=\lambda_{0}+\lambda_{01}R+\lambda_{10}Q\text{.}$$

Plugging (103) along with (4), (5), (102) provides us a polynomial of Q,R and $R^{\prime }$. After collecting all terms with the same exponents, we set the resulting coefficients equal to zero. This yields a system of equations that leaves us with the result:

$$\lambda_{0}=\lambda_{10}=0,\lambda_{01}=\pm \sqrt{-\frac{\chi \left(\alpha \left(m+5\right)-4\beta \right)}{a{\left(m+2\right)}^{2}}},k=\left(m+1\right)\sqrt{-\frac{b_{3}\left(\alpha \left(m+5\right)-4\beta \right)}{2a^{2}{\left(m+2\right)}^{3}\left(3m+5\right)}}\text{,}$$

(104) $$\omega =\frac{8b_{3}\left(\alpha -\beta \right)\left(\alpha \left(m+5\right)-4\beta \right)}{a^{2}{\left(m+2\right)}^{3}\left(3m+5\right)}+\gamma ,b_{1}=0\text{.}$$

Putting (104) with the aid of (6), (103), the nonlinear waveform is recovered as

$$q\left(x,t\right)={\left\{\pm \frac{4c\sqrt{-\frac{\chi \left(\alpha \left(m+5\right)-4\beta \right)}{a{\left(m+2\right)}^{2}}}}{4c^{2}\;\exp\left[\left(m+1\right)\sqrt{-\frac{b_{3}\left(\alpha \left(m+5\right)-4\beta \right)}{2a^{2}{\left(m+2\right)}^{3}\left(3m+5\right)}}\;x\right]+\chi \;\exp\left[-\left(m+1\right)\sqrt{-\frac{b_{3}\left(\alpha \left(m+5\right)-4\beta \right)}{2a^{2}{\left(m+2\right)}^{3}\left(3m+5\right)}}\;x\right]}\right\}}^{\frac{2}{m+1}}$$

(105) $$\times e^{i\left(\left\{\frac{8b_{3}\left(\alpha -\beta \right)\left(\alpha \left(m+5\right)-4\beta \right)}{a^{2}{\left(m+2\right)}^{3}\left(3m+5\right)}+\gamma \right\}t+\theta_{0}\right)}\text{.}$$

Assuming $\chi =\pm 4c^{2}$ in solution (105) reveals the quiescent singular and bright solitons

$$q\left(x,t\right)={\left\{\sqrt{\frac{\alpha \left(m+5\right)-4\beta }{a{\left(m+2\right)}^{2}}}\mathrm{csch}\left[\left(m+1\right)\sqrt{-\frac{b_{3}\left(\alpha \left(m+5\right)-4\beta \right)}{2a^{2}{\left(m+2\right)}^{3}\left(3m+5\right)}}\;x\right]\right\}}^{\frac{2}{m+1}}$$

$$\times e^{i\left(\left\{\frac{8b_{3}\left(\alpha -\beta \right)\left(\alpha \left(m+5\right)-4\beta \right)}{a^{2}{\left(m+2\right)}^{3}\left(3m+5\right)}+\gamma \right\}t+\theta_{0}\right)}\text{,}$$

and

$$q\left(x,t\right)={\left\{\sqrt{-\frac{\alpha \left(m+5\right)-4\beta }{a{\left(m+2\right)}^{2}}}\mathrm{sech}\left[\left(m+1\right)\sqrt{-\frac{b_{3}\left(\alpha \left(m+5\right)-4\beta \right)}{2a^{2}{\left(m+2\right)}^{3}\left(3m+5\right)}}\;x\right]\right\}}^{\frac{2}{m+1}}$$

(106) $$\times e^{i\left(\left\{\frac{8b_{3}\left(\alpha -\beta \right)\left(\alpha \left(m+5\right)-4\beta \right)}{a^{2}{\left(m+2\right)}^{3}\left(3m+5\right)}+\gamma \right\}t+\theta_{0}\right)}\text{,}$$

where

$$b_{3}\left(\alpha \left(m+5\right)-4\beta \right)<0\text{.}$$

**Fig. 11:**
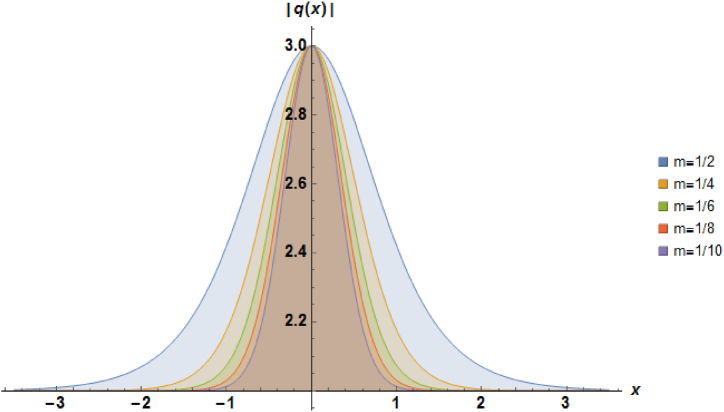
Characteristics of a stationary bright soliton profile.

Plots of the soliton described by equation (106) with β=−1, α=1, $b_{3}=-1$ and a=1 are displayed in Fig. 11.

## 4 Conclusions

The current paper retrieved stationary optical solitons from the CGLE for a dozen forms of nonlinear refractive index structures. The results of the paper thus send a strong message to electronics and telecommunication engineers that if the CD is rendered to be nonlinear, soliton transmission across intercontinental distances is halted thus bringing the internet communication across the globe to a standstill. Such a catastrophic consequence can be avoided by making absolutely sure that CD is never rendered to be nonlinear during its transmission. The future of the project also holds strong with these preliminary results. The model will be next extended with dispersion flattened fibers and also with differential group delay. The findings from these studies will be subsequently compared and incorporated into the previously published works cited in Refs. [[13], [14], [15], [16], [17], [18], [19], [20]].

## Author contribution statement

Ahmed H. Arnous, Anjan Biswas: Conceived and designed the experiments; Wrote the paper. Yakup Yıldırım: Performed the experiments; Wrote the paper. Luminita Moraru, Simona Moldovanu: Analyzed and interpreted the data; Wrote the paper. Abdulah A. Alghamdi: Contributed reagents, materials, analysis tools or data; Wrote the paper.

## Funding statement

This research did not receive any specific grant from funding agencies in the public, commercial, or not-for-profit sectors.

## Data availability

No data was used for the research described in the article.

## Declaration of competing interest

The authors declare no competing interests.

## References

[1] Adem A.R., Ntsime B.P., Biswas A., Khan S., Alzahrani A.K., Belic M.R. (2021). Stationary optical solitons with nonlinear chromatic dispersion for Lakshmanan–Porsezian–Daniel model having Kerr law of nonlinear refractive index. Ukr. J. Phys. Opt..

[2] Adem A.R., Ntsime B.P., Biswas A., Ekici M., Yildirim Y., Alshehri H.M. (2022). Implicit quiescent optical solitons with complex Ginzburg–Landau equation having nonlinear chromatic dispersion. J. Optoelectron. Adv. Mater..

[3] Ekici M. (2022). Stationary optical solitons with complex Ginzburg–Landau equation having nonlinear chromatic dispersion and Kudryashov's refractive index structures. Phys. Lett..

[4] Ekici M. (2023). Stationary optical solitons with Kudryashov's quintuple power law nonlinearity by extended Jacobi's elliptic function expansion. J. Nonlinear Opt. Phys. Mater..

[5] Hong W.P. (2008). Existence conditions for stable stationary solitons of the cubic–quintic complex Ginzburg–Landau equation with a viscosity term. Z. Naturforsch..

[6] Kudryashov N.A. (2022). Stationary solitons of the model with nonlinear chromatic dispersion and arbitrary refractive index. Optik.

[7] Kydryashov N.A. (2022). Stationary solitons of the generalized nonlinear Schrödinger equation with nonlinear dispersion and arbitrary refractive index. Appl. Math. Lett..

[8] Sonmezoglu A. (2022). Stationary optical solitons having Kudryashov's quintuple power law nonlinearity by extended *G’/G*–expansion. Optik.

[9] Yalci A.M., Ekici M. (2022). Stationary optical solitons with complex Ginzburg–Landau equation having nonlinear chromatic dispersion. Opt. Quant. Electron..

[10] Yan Z. (2006). Envelope compactons and solitary patterns. Phys. Lett..

[11] Yan Z. (2006). Envelope compact and solitary pattern structures for the *GNLS*(m,n,p,q) equations. Phys. Lett..

[12] Zayed E.M.E., Alngar M.E.M., Biswas A., Ekici M., Khan S., Alshomrani A.S. (2021). Pure–cubic optical soliton perturbation with complex Ginzburg–Landau equation having a dozen nonlinear refractive index structures. J. Commun. Technol. Electron..

[13] Shehata M.S.M., Rezazadeh H., Zahran E.H.M., Tala–Tebue E., Bekir A. (2019). New optical soliton solutions of the perturbed Fokas–Lenells equation. Commun. Theor. Phys..

[14] Rezazadeh H. (2018). New solitons solutions of the complex Ginzburg-Landau equation with Kerr law nonlinearity. Optik.

[15] Eslami M., Rezazadeh H. (2016). The first integral method for Wu–Zhang system with conformable time-fractional derivative. Calcolo.

[16] Hosseini K., Hincal E., Mirzazadeh M., Salahshour S., Obi O.A., Rabiei F. (2023). A nonlinear Schrödinger equation including the parabolic law and its dark solitons. Optik.

[17] Hosseini K., Hincal E., Salahshour S., Mirzazadeh M., Dehingia K., Nath B.J. (2023). On the dynamics of soliton waves in a generalized nonlinear Schrödinger equation. Optik.

[18] Hosseini K., Hincal E., Baleanu D., Obi O.A., Salahshour S. (2023). Non-singular multi-complexiton wave to a generalized KdV equation. Nonlinear Dynam..

[19] Madhukalya B., Das R., Hosseini K., Baleanu D., Hincal E. (2023). Effect of ion and negative ion temperatures on KdV and mKdV solitons in a multicomponent plasma. Nonlinear Dynam..

[20] Kalita J., Das R., Hosseini K., Baleanu D., Salahshour S. (2023). Solitons in magnetized plasma with electron inertia under weakly relativistic effect. Nonlinear Dynam..

